# LncDARS‐AS1 Regulates ATP1A1 Stability and Enhances Na^+^/K^+^ ATPase Activity to Promote Osteosarcoma Metastasis

**DOI:** 10.1002/advs.202503486

**Published:** 2025-07-15

**Authors:** Mingxian Xu, Jiatian Wei, Xiaoyu Feng, Qinkai Zhang, Jian Chen, Xinyue Wang, Xiudan Zhan, Bing Lu, Weitang Guo, Mingzhe Cheng, Renxuan Huang, Shao Xu, Changye Zou

**Affiliations:** ^1^ Department of Musculoskeletal Oncology The First Affiliated Hospital of Sun Yat‐sen University Guangzhou Guangdong 510080 China; ^2^ Guangdong Provincial Key Laboratory of Orthopedics and Traumatology The First Affiliated Hospital Sun Yat‐sen University Guangzhou 510080 China; ^3^ Center for Stem Cell Biology and Tissue Engineering Key Laboratory for Stem Cells and Tissue Engineering Ministry of Education Sun Yat‐sen University Guangzhou 510000 China; ^4^ Medical Research Institute Guangdong Provincial People's Hospital (Guangdong Academy of Medical Sciences) Southern Medical University Guangzhou 510080 China; ^5^ Department of Stomatology The Third Affiliated Hospital of Southern Medical University Guangzhou Guangdong 510630 China

**Keywords:** ATP1A1, digoxin, LncDARS‐AS1, metastasis, Na/K‐ATPase, osteosarcoma, tumor therapy, ubiquitination

## Abstract

Osteosarcoma, the most prevalent malignant bone tumour in children and adolescents, exhibits aggressive pulmonary metastasis and poor prognosis. This study identifies LncDARS‐AS1 as a key regulator of metastasis via modulation of ATP1A1, the catalytic subunit of Na⁺/K⁺ ATPase (NKA). Transcriptomic analyses, validated by qPCR in 217 osteosarcoma RNA samples, reveal that LncDARS‐AS1 is significantly upregulated in metastatic lesions and associated with adverse clinical outcomes. Functional assays confirm that silencing LncDARS‐AS1 suppresses osteosarcoma proliferation and metastasis in vitro and in vivo. Mechanistically, LncDARS‐AS1 directly binds ATP1A1, preventing its interaction with the UBQLN4 and subsequent proteasomal degradation, thereby enhancing NKA activity. Protein‐RNA interactions were validated using ChIRP, mass spectrometry, molecular docking, and molecular dynamics simulations. Functional NKA activity was assessed using ion‐sensitive fluorescent indicators and enzymatic assays. Additionally, digoxin, a cardiac glycoside targeting NKA, effectively inhibited tumour growth and metastasis at clinically safe concentrations. These findings uncover a novel LncDARS‐AS1/ATP1A1 axis that promotes osteosarcoma metastasis through inhibition of ubiquitin‐mediated degradation and provide a rationale for repurposing digoxin in osteosarcoma therapy. ATP1A1 emerges as a promising target for anti‐metastatic intervention.

## Graphical abstract

This study identifies LncDARS‐AS1 as a novel oncogenic regulator that stabilizes ATP1A1 and enhances Na⁺/K⁺ ATPase activity, promoting osteosarcoma metastasis. Targeting the LncDARS‐AS1/ATP1A1/NKA axis, including pharmacological inhibition with digoxin, offers promising opportunities for the development of new therapeutic strategies against osteosarcoma.

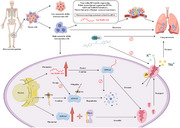

## 1 Introduction

Osteosarcoma is the most common primary malignant bone tumor, primarily affecting children and adolescents, and is known for its aggressive biological behavior.^[^ ^1^ ^]^ The aggressive nature of osteosarcoma, marked by early pulmonary metastasis, remains a key cause of treatment failure and high mortality.^[^ ^2^ ^]^ Despite significant advancements in therapeutic strategies and a deeper understanding of its pathogenesis, the 5‐year survival rate for patients without clinically detectable metastatic disease has improved from 30% to over 70%.^[^ ^3^ ^]^ Controlling metastasis remains a major challenge in improving osteosarcoma survival. The underlying molecular mechanisms are poorly understood, highlighting the need to identify key regulatory factors to inform more effective diagnostic and therapeutic strategies.

Long non‐coding RNAs (LncRNAs) are far more abundant than protein‐coding genes. Once regarded as mere byproducts of transcription with limited regulatory roles^[^ ^4^, ^5^ ^]^, LncRNAs play essential roles in diverse biological processes, including the regulation of tumor initiation and progression in osteosarcoma and other malignancies.^[^ ^4^, ^5^, ^6^, ^7^ ^]^ While existing studies suggest that LncRNAs modulate gene expression through diverse mechanisms in osteosarcoma, their exact role in promoting metastasis and tumor progression remains unclear.^[^ ^8^, ^9^ ^]^

LncDARS‐AS1 is markedly overexpressed in multiple myeloma and has been closely linked to poor clinical outcomes. Mechanistically, LncDARS‐AS1 binds to RBM39 and disrupts its interaction with the E3 ubiquitin ligase RNF147, thereby preventing RBM39 ubiquitination and degradation, ultimately facilitating malignant progression.^[^ ^10^ ^]^ Broad expression of LncDARS‐AS1 has been observed across various cancer types, with elevated levels consistently associated with unfavorable prognosis. In particular, LncDARS‐AS1 directly interacts with the activation domain of PACT, impeding the PACT–PKR interaction, reducing PKR activation and subsequent eIF2α phosphorylation, and thereby suppressing apoptosis in tumor cells.^[^ ^11^ ^]^ In clear cell renal cell carcinoma (ccRCC), LncDARS‐AS1 is significantly upregulated and correlates with poor survival; it promotes tumor progression via transcriptional upregulation of DARS.^[^ ^12^ ^]^ In triple‐negative breast cancer (TNBC), LncDARS‐AS1 is highly enriched in tumor tissues and cell lines, with its expression positively correlated with advanced clinical stage. Functional studies have demonstrated that LncDARS‐AS1 enhances the migratory and invasive capacity of TNBC cells through activation of the NF‐κB/STAT3 signaling pathway.^[^ ^13^ ^]^

Building on our previous findings, which included paired non‐coding RNA probe sequencing, whole transcriptome sequencing (WTS), and other RNA sequencing analyses in osteosarcoma patients^[^ ^14^ ^]^, LncDARS‐AS1 was identified as significantly upregulated in osteosarcoma patients with pulmonary metastasis, correlating with poor prognosis. This finding was validated through large‐scale tissue analysis. Functional studies demonstrated that silencing LncDARS‐AS1 significantly inhibited osteosarcoma cell proliferation and metastasis, both in vitro and in vivo. Mechanistically, LncDARS‐AS1 interacts with the ATP1A1‐UBQLN4 complex, preventing the ubiquitination and subsequent degradation of ATP1A1, thereby enhancing Na^+^/K^+^ ATPase (NKA) activity and promoting tumor growth and metastasis. Additionally, the study provides the first evidence supporting the potential clinical use of digoxin, a targeted Na^+^/K^+^ ATPase (NKA) inhibitor, in suppressing osteosarcoma metastasis. Therefore, this study aims to explore the molecular mechanism by which LncDARS‐AS1 promotes osteosarcoma metastasis through the regulation of ATP1A1.

## 2 Experimental Section

### 2.1 Clinical Sample Preparation

Between January 2015 and December 2019, 217 pairs of primary osteosarcoma tissues and corresponding adjacent non‐tumor tissues were collected from the First Affiliated Hospital of Sun Yat‐sen University, Guangzhou, China. Participants were included based on the following criteria: 1) undergoing curative surgical resection, 2) availability of tumor tissue specimens, and 3) availability of complete clinicopathological and longitudinal follow‐up data. Adjacent non‐tumor tissues were collected at least 5 cm away from the primary tumor site. Tissue specimens were immediately snap‐frozen in liquid nitrogen to preserve DNA, RNA, and protein integrity for subsequent extraction. The clinicopathological characteristics of the study cohort are detailed in **Table** **1**. This study was conducted in accordance with the ethical standards set by the Ethical Committee of the First Affiliated Hospital of Sun Yat‐sen University, under approval ID [2023]547. Of the 217 patients initially screened, 117 were excluded due to preoperative chemotherapy‐induced tumor necrosis, which compromised RNA integrity, and 6 were excluded due to incomplete clinical data, resulting in 94 patients included in the final analysis. Excluding patients with tumor necrosis was essential to minimize confounding from chemotherapy‐induced tissue alterations that could compromise the accuracy of gene and protein expression analyses. These exclusion criteria might limit the generalizability of the findings due to the rarity of osteosarcoma. However, building on previous work that included paired non‐coding RNA probe sequencing, whole transcriptome sequencing (WTS), and other RNA sequencing analyses in osteosarcoma patients, the integration of data from 94 samples provided strong evidence for the reliability of the gene screening approach, ensuring the validity of the results despite the reduced sample size.

**Table 1:** Patient Characteristics.

Variable	Total
Initial patient	217
Excluded patient	123
Included patient	94
Sex (no. [%])	
Male	51 (54.26%)
Female	43(45.74%)
Age of diagnosis (no.)(years)	
Mean	18.64
Std. dev.	10.85
Median	16.00
Range	8‐71
Enecking stage (no. [%])	
II	82 (87.23%)
III	12(12.77%)
Relapse (no. [%])	11 (11.70%)
Metastasis (no. [%])	28(29.79%)
Follow‐up time(no.[%])(months)	
Follow‐up cases	94 (100%)
Mean	47.65
Median	47.5
Range	9‐99
Survival status (no. [%])	
Survival	49(52.13%)
Death	45(47.87%)

### 2.2 Extraction and Processing of Gene Expression Data

Five gene expression profiling datasets (GSE14359, GSE14827, GSE21257, GSE32981, GSE42352) were selected and downloaded from the Gene Expression Omnibus (GEO) database (https://www.ncbi.nlm.nih.gov/geo/). These datasets include 17 normal human cell samples and 114 osteosarcoma samples. Gene expression and clinical data for osteosarcoma patients were also retrieved from the Therapeutically Applicable Research to Generate Effective Treatments (TARGET) database. Survival differences between groups were evaluated using the log‐rank test, and data processing was performed with R software (version 4.0.3).

### 2.3 Cell Lines and Transfection

The osteosarcoma (OS) cell lines (143B, MNNG/HOS, HOS, U_2_OS/MTX^300^, U_2_OS, G292, SJSA‐1, MG63, and SaOS‐2) and the normal human osteoblast cell line (hFOB1.19) used in this study are summarized in Table S1 (Supporting Information). OS cells were cultured in medium supplemented with 10% fetal bovine serum (FBS; Gibco) at 37 °C in a humidified atmosphere containing 5% CO_2_. In contrast, hFOB1.19 cells were maintained at 35 °C in medium containing 15% FBS, according to the supplier's recommended conditions. shRNA constructs targeting DARS‐AS1, ATP1A1, UBQLN4, and a control shRNA were cloned into the pLKO.1 vector. Full‐length DARS‐AS1 was amplified by PCR from cDNA, and truncated variants were generated through molecular cloning of the full‐length construct, followed by insertion into the pCDH‐CMV‐MCS‐EF1‐Puro vector. Full‐length ATP1A1 was HA‐tagged upstream of the start codon and cloned into the pcDNA3.1 vector to generate HA‐tagged constructs. Truncated versions of ATP1A1 were prepared by deleting specific domains (Table S1, Supporting Information). All constructs were sequenced for integrity by IGE Biotechnology (Guangzhou, China) and transfected into cells using Lipofectamine 3000 (Invitrogen, USA) according to the manufacturer's instructions. Stable cell lines were selected with puromycin.

### 2.4 Quantitative Real‐Time PCR

RNA was isolated using Trizol (Invitrogen, USA), and its purity and concentration were assessed via UV spectrophotometry. cDNA was synthesized by reverse transcription using a Roche kit (Basel, Switzerland), followed by quantitative Real‐Time PCR with the SYBR Green system (Applied Biosystems, USA) to quantify gene expression. Relative expression levels were calculated using the 2^−ΔΔCt^ method. Primers were obtained from RiboBio (Guangzhou, China) (Table S1, Supporting Information).

### 2.5 Cell Viability and Cell Proliferation Assay

Cell viability was assessed using the Cell Counting Kit‐8 (CCK‐8) assay (Dojindo, Kumamoto, Japan). Transfected cells (3×10^^3^ cells/well) were plated onto 96‐well plates and measured at 0, 24, 48, and 72 h after treatment, according to the manufacturer's instructions. Absorbance at 450 nm was recorded as an indicator of cell viability. All experiments were performed in quadruplicate.

For the colony formation assay, cells (500 cells/well) were plated into 6‐well plates and cultured for 2 weeks in medium supplemented with 10% FBS, with medium replaced every 4 days. After 10 days, cells were fixed with 4% paraformaldehyde (Phygene, Fu‐Zhou, China) for 15 min to preserve cell morphology, followed by staining with 1% crystal violet (Beyotime, Nantong, China). Cell clones were counted and analyzed.

### 2.6 Transwell Assay

After appropriate treatments, cells were seeded in the upper chamber of a Transwell insert at a density of 2×10^^4^ cells/well in 100 µL of serum‐free medium. The lower chamber contained medium with 20% FBS as a chemoattractant. Migration assays were conducted using inserts without Matrigel, while invasion assays were performed using 8 µm inserts (Corning, #3422, USA) coated with Matrigel (BD Biosciences, NJ, USA). After 24 h of incubation, cells and excess Matrigel in the upper chamber were removed. Cells were fixed with 4% paraformaldehyde (Phygene, Fu‐Zhou, China) for 25 min and stained with crystal violet (Beyotime, Nantong, China) for 10 min. Migratory and invasive cells were photographed and quantified in five randomly selected fields using an optical microscope.

### 2.7 Western Blotting (WB) Analysis and Subcellular Fractionation

Protein content in the lysates was assessed using the BCA protein assay (Beyotime, Shanghai, China). Nuclear and cytoplasmic fractions from 143B and U_2_OS/MTX^300^ cells were prepared according to the manufacturer's instructions (PARIS Kit, Thermo Fisher). The cytoplasmic and nuclear fractions were analyzed for both protein and RNA content. Proteins were denatured by incubating in loading buffer at 95 °C for 10 min, and ATP1A1 protein was denatured at 50 °C. Proteins were separated using 10% SDS‐PAGE and transferred to 0.22 µm PVDF membranes (Millipore, Billerica, USA). Membranes were then incubated with specific antibodies at 4 °C overnight. Horseradish peroxidase‐linked secondary antibodies were added and incubated at 37 °C for 1 h. After washing the membranes three times with TBST, immunoreactive bands were visualized using an ECL Kit (Thermo Fisher). The gray value of the bands was quantified using an image analyzer to indicate protein expression levels.

### 2.8 Reagents and Antibodies

Antibodies against ATP1A1 (14418‐1‐AP), α‐Tubulin (11224‐1‐AP), and Ki67 (28074‐1‐AP) were purchased from Proteintech. The β‐actin antibody (4967L) was purchased from Cell Signaling Technology (Beverly, MA, USA). The UBQLN4 antibody was obtained from Novus (NBP3‐14725, USA) and SAB (29463‐1, USA). The anti‐Ubiquitin antibody (P4D1) was from Santa Cruz Biotechnology (Santa Cruz, CA, USA). Horseradish peroxidase‐conjugated anti‐mouse IgG and anti‐rabbit IgG were obtained from Millipore (Bedford, MA, USA). Media, fetal bovine serum (FBS), and tissue culture supplies were obtained from GIBCO (Grand Island, NY, USA). Leupeptin (HY‐18234), chloroquine (HY‐17589A), MG132 (HY‐13259), digoxin (HY‐B1049), Cycloheximide (CHX) (HY‐12320), and Eeyarestatin I (HY‐110078) were obtained from MedChemExpress (MCE). All chemicals were purchased from Sigma (St. Louis, MO, USA).

### 2.9 Luciferase Assays

Osteosarcoma (OS) cells were seeded in 96‐well plates (5,000 cells per well) and co‐transfected with 100 ng of the psicheck2 Luciferase vector, containing the 3' UTR of target genes, and 200 nm of DARS‐AS1 inhibitor, DARS‐AS1 mimics, or negative control mimics (Inhibitor control/Mimic control). The experimental plasmids were constructed by Zhongchen Medical Laboratory (Guangzhou, China). Forty‐eight hours post‐transfection, the Dual‐Luciferase Reporter Assay (Promega) was performed according to the manufacturer's instructions.

### 2.10 Chromatin Isolation by RNA Purification (ChIRP) Combined with Mass Spectrometry (MS)/Western Blot (WB)

ChIRP‐MS experiments were performed with 143B cells. Cell harvesting and disruption, along with ChIRP analysis, were conducted according to the manufacturer's instructions (RiboBio, Guangzhou, China).^[^ ^15^ ^]^ A set of three 3′‐biotinylated antisense DNA probes, each 20 nucleotides in length, was designed to target spatially distinct and structurally accessible regions of the LncDARS‐AS1 transcript, based on predicted secondary structure and transcript conservation. A non‐targeting probe was used as a negative control. Probes were captured using streptavidin‐coated magnetic beads and washed five times with wash buffer at 37 °C. The retrieved proteins were subsequently analyzed by liquid chromatography‐tandem mass spectrometry (LC‐MS/MS) (Wininnovate Bio, Shenzhen, China) and Western Blot. Detailed probe sequences and design information are provided in Table S1 (Supporting Information) (DARS‐AS1 Probe [ChIRP]).

### 2.11 Co‐Immunoprecipitation Assay (Co‐IP)

Cell lysates were prepared with a lysis buffer, and immunoprecipitation was performed overnight at 4 °C using antibodies (ATP1A1, ubiquitin, UBQLN4, or IgG isotype control). Magnetic beads (Thermo Fisher Scientific, #26162, MA, USA) were added to the lysates for incubation at 4 °C for 4 h. The proteins were analyzed by Western blot or mass spectrometry. To determine the ATP1A1 binding sites in DARS‐AS1, 143B cells were transfected with wild‐type or mutant pcDNA3.1‐DARS‐AS1 plasmids (Table S1, Supporting Information). 143B cell lysates transfected with HA‐ATP1A1 (Table S1, Supporting Information) were used for immunoprecipitation with anti‐HA beads (MCE, HY‐K0201A). Western blot analysis was performed to examine coimmunoprecipitated proteins.

### 2.12 Turnover Assays

Stably transfected osteosarcoma (OS) cells with shDARS‐AS1 or control (shCtrl) were seeded in 6‐well plates and cultured for 24 h. Cycloheximide (CHX) was added to a final concentration of 200 µg mL^−1^ to inhibit new protein synthesis. The cells were harvested at the indicated time points following CHX treatment. Western blot analysis was performed to measure the protein levels of ATP1A1 and α‐Tubulin.

### 2.13 Animal Experiments

All BALB/c nude mice (4–6 weeks, 20–25g, female) were maintained under pathogen‐free conditions (Ruiye Model Animal Biotechnology, Guangzhou, China). All procedures were approved by the Guidance of Institutional Clinical Research and Animal Trials (Application ID: [2023]016) of the First Affiliated Hospital of Sun Yat‐sen University and conducted in accordance with the Guide for the Care and Use of Laboratory Animals. Approximately 5×10^^5^ 143B cells, suspended in 20 µL PBS, were injected into the tibial medulla to establish an osteosarcoma orthotopic mouse model. Osteosarcoma growth in vivo was monitored after 10 days using an in vivo imaging system (IVIS, Xenogen) under isoflurane anesthesia. At the end of the experiment, mice were euthanized in a chamber using 100% CO_2_. For in vivo validation of gene expression (DARS‐AS1 and ATP1A1), nude mice were randomly divided into three groups (n = 10 per group): shCtrl, sh#1, and sh#2. For the pharmacological intervention study, animals were randomized into three groups (n = 10 per group) and treated with either vehicle control (saline), low‐dose digoxin (0.1 mg kg^−1^), or high‐dose digoxin (0.2 mg kg^−1^). Digoxin or saline was administered via intraperitoneal injection every other day for a total duration of 4 weeks. At the end of the treatment period, all mice were euthanized under anesthesia, and tumors were surgically excised for analysis. All animal experiments included 10 mice per group, and group assignment, treatment, and analysis were performed under rigorously controlled and randomized conditions to ensure reliability and reproducibility.

### 2.14 Measurement of Intracellular Sodium and Potassium Concentration

Osteosarcoma cells were plated into 96‐well culture plates (Corning, LG18‐301C‐6) and maintained in complete medium overnight. The following day, the medium was replaced with low serum (1%) and incubated for 45 min. Cells were then loaded with 10 µm PBFI‐AM (Potassium Binding Fluorescent Indicator, Abcam, ab142804) or 10 µm SBFI‐AM (Sodium Binding Fluorescent Indicator, Abcam, ab142800) in the presence of 0.1% Pluronic F‐127 (Sigma, P2443‐250G) to enhance dye solubility. After 100 min, excess dye was removed by washing with low serum medium. A High Intelligent and Sensitive SIM System was used to acquire fluorescence signals by capturing five images: one with an excitation wavelength of 340 nm and an emission wavelength of 540 nm, and another with an excitation wavelength of 380 nm and an emission wavelength of 540 nm, at 0 and 4 h after the addition of PBFI or SBFI. Fluorescence excitation ratios at 340 and 380 nm, measured at 500 nm emission using a microplate reader, were used to estimate intracellular K^+^ or Na^+^ concentrations. Results were corrected for background fluorescence and expressed as a percentage of the untreated control.^[^ ^16^, ^17^ ^]^

### 2.15 Na⁺/K⁺‐ATPase Activity Measurement

Na⁺/K⁺‐ATPase activity in osteosarcoma cells with varying gene expression levels was measured using the CheKine Na⁺/K⁺‐ATPase Activity Colorimetric Assay Kit (KTB1800, Abbkine, Wuhan, China), following the manufacturer's instructions. Results were calculated based on the cell count, with one unit of enzyme activity defined as the amount of ATP hydrolyzed to produce 1 µmol of inorganic phosphate per 10,000 cells per hour.

### 2.16 Immunohistochemical (IHC) Staining and Hematoxylin‐Eosin (HE) Staining

ATP1A1 protein levels were assessed by IHC. Paraffin‐embedded tissue sections were baked, dewaxed, and hydrated before undergoing antigen retrieval and blocking. The sections were then incubated overnight at 4 °C with an anti‐ATP1A1 antibody. The following day, secondary antibodies were applied, and staining was visualized using the GTVision III Detection System/Mo&Rb (GeneTech, China/Servicebio, Wuhan). Immunohistochemical staining was evaluated based on both staining intensity and the proportion of positive tumor cells. Staining intensity was scored as 0 (no staining), 1 (weak), 2 (moderate), or 3 (strong), while the percentage of positive cells was scored as 0 (<5%), 1 (5%–25%), 2 (26%–50%), 3 (51%–75%), or 4 (>75%). An immunoreactive score (IRS) was then calculated by multiplying the two values, yielding a range from 0 to 12. All slides were independently assessed by two experienced pathologists blinded to clinical data, with discrepancies resolved by consensus. For survival analysis, patients were stratified into high and low ATP1A1 expression groups using the median IRS as the cutoff. Kaplan–Meier survival curves were generated using overall survival as the endpoint. Hematoxylin‐eosin staining was performed as previously described.^[^ ^18^ ^]^

### 2.17 Molecular Docking and Molecular Dynamics (MD) Trajectories Analysis

Protein sequences and AlphaFold2 structures^[^ ^19^ ^]^ were obtained from the UniProt database.^[^ ^20^ ^]^ To improve modeling accuracy, B‐factors and disordered regions were removed from the models for simulation. Molecular docking was performed using Smina.^[^ ^21^ ^]^ MD simulations were carried out using GROMACS.^[^ ^22^ ^]^ Electrostatic interactions were handled using particle mesh Ewald (PME) under elastic simulation with the Verlet and conjugate gradient (CG) schemes. Energy minimization was performed using the steepest descent method with a maximum of 50,000 steps. Coulomb and van der Waals interactions were both cut off at 1.4 nm. Simulations were run for 100 ns under room conditions. LINCS constrained hydrogen bonds with a 2‐fs integration step. PME was used for long‐range electrostatics with a 1.2 nm cutoff, and non‐bonded interactions were cut off at 10 Å. Temperature was maintained at 300 K using V‐rescale, and pressure at 1 bar with Berendsen. NVT and NPT equilibration were performed for 30 ps at 300 K. Trajectory analysis included RMSD, Rg, RMSF, and SASA.

### 2.18 Statistical Analysis

All experiments were repeated independently at least three times. Statistical analysis was performed using SPSS software 26.0 for Windows (SPSS Inc., Chicago, IL, USA) or GraphPad Prism 9 to assess differences between experimental groups. Data are presented as mean±standard deviation. Differences between groups were assessed using the student's t‐test, Wilcoxon test, or Chi‐square test. Disease‐free survival (DFS) and overall survival (OS) were determined using the Kaplan–Meier method, with group comparisons based on the expression levels of LncDARS‐AS1 and ATP1A1 (classified as high or low expression based on median expression levels). Intergroup differences were evaluated using the log‐rank test. P values below 0.05 were considered statistically significant.

### 2.19 Ethics Statement

All procedures and data analyses in the patient were approved by the ethics committee of The First Affiliated Hospital of Sun Yat‐sen University. The use of human tissues in this study was approved by the Ethics Committee of The First Affiliated Hospital of Sun Yat‐sen University (Approval ID: [2023]547). The animal protocol was approved by the Animal Ethics Committee of The First Affiliated Hospital of Sun Yat‐sen University (Application ID: [2023]016).

## 3 Results

### 3.1 LncDARS‐AS1 Upregulation is Associated with Osteosarcoma Metastasis and Poor Prognosis

**Figure 1:**
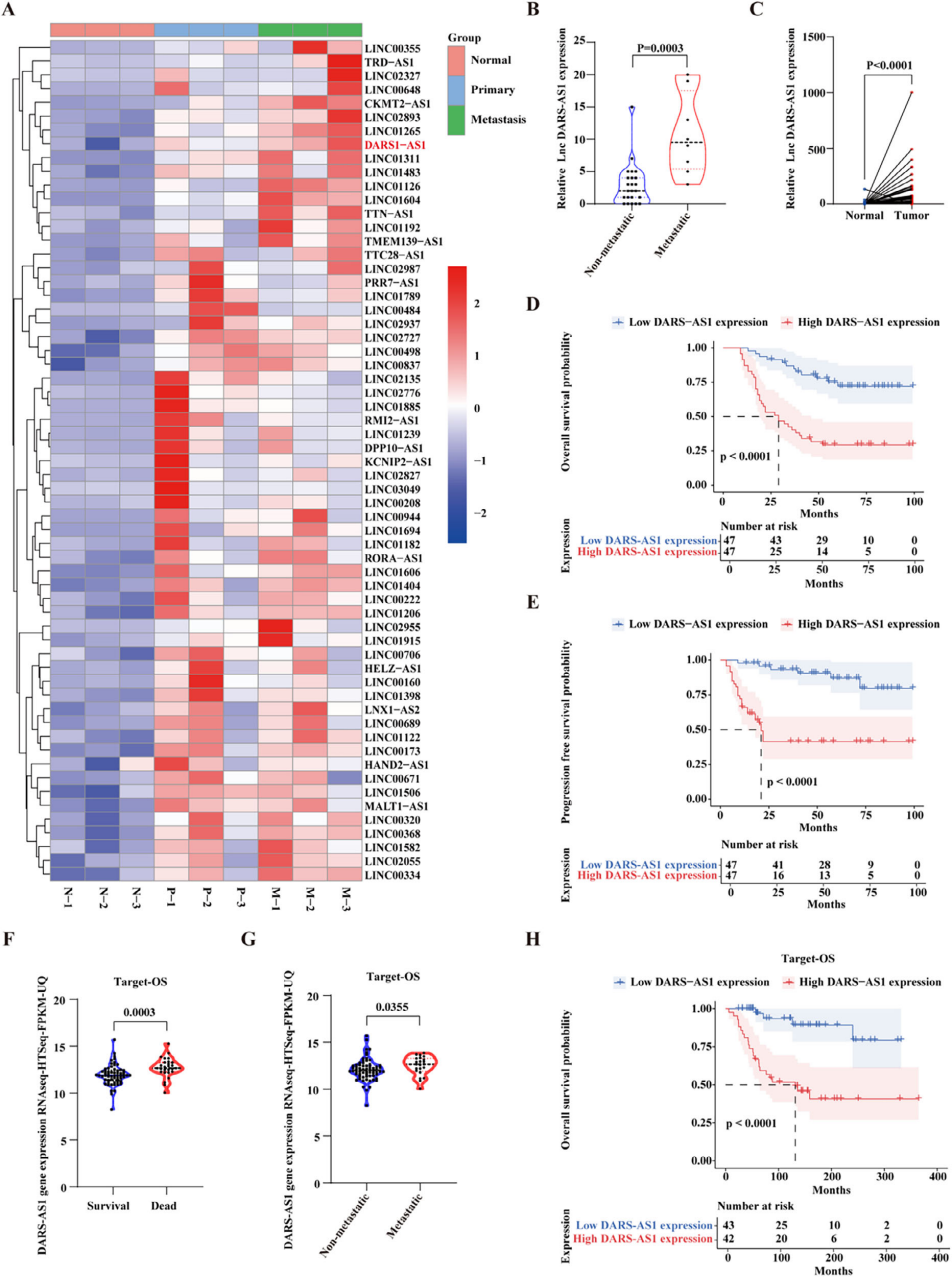
Expression and prognostic significance of LncDARS‐AS1 in osteosarcoma. A) Comparative analysis of non‐coding RNA expression in lung metastasis tissues from three osteosarcoma patients demonstrated a significant upregulation of LncDARS‐AS1 in lung metastasis compared to normal adjacent and primary tumor tissues. B) Whole transcriptome sequencing (WTS) analysis of 31 osteosarcoma patients (8 with metastasis, 23 without) demonstrated higher LncDARS‐AS1 expression in patients with lung metastasis. C) RT‐PCR analysis of 94 paired osteosarcoma and adjacent normal tissue samples demonstrated a significantly higher expression of LncDARS‐AS1 in osteosarcoma tissues compared to adjacent normal tissues. D) Kaplan–Meier analysis of overall survival (OS) in osteosarcoma patients stratified by LncDARS‐AS1 expression levels. High LncDARS‐AS1 expression was associated with significantly poorer OS. E) Kaplan–Meier analysis of progression‐free survival (PFS) in osteosarcoma patients stratified by LncDARS‐AS1 expression levels. High LncDARS‐AS1 expression was associated with significantly poorer PFS. F) Analysis of the TARGET‐OS dataset showed significantly higher LncDARS‐AS1 expression in patients who died compared to those who survived (p = 0.0003). G) Analysis of the TARGET‐OS dataset revealed significantly higher LncDARS‐AS1 expression in patients who developed metastasis compared to those without metastatic disease (p = 0.0355). H) Analysis of the TARGET‐OS dataset indicated that higher LncDARS‐AS1 expression was associated with significantly decreased overall survival (OS) compared to lower expression levels.

To investigate the oncogenic role of lncRNAs in osteosarcoma progression, a comparative analysis was performed using non‐coding RNA probe data from three patients with lung metastases, with three samples each from adjacent normal tissues, primary tumor tissues, and lung metastasis tissues. LncDARS‐AS1 was significantly upregulated in the lung metastasis samples (**Figure** **1**A). Further analysis of gene expression data from 31 patients (8 with metastasis, 23 without metastasis) subjected to whole transcriptome sequencing (WTS) revealed higher expression levels of LncDARS‐AS1 in the patients with lung metastases (Figure 1B).

To confirm the elevated expression of LncDARS‐AS1 in osteosarcoma tissues, RNA from 217 paired tissue samples was analyzed by RT‐PCR. A total of 94 samples were included for statistical analysis. The results showed that the expression of LncDARS‐AS1 was significantly higher in osteosarcoma tissues compared to adjacent normal tissues (Figure 1C). Notably, patients with high LncDARS‐AS1 expression had significantly lower OS and PFS compared to those with lower expression (Figure 1D,E).

Additionally, RNA sequencing data and clinical records from the TARGET‐OS dataset were analyzed, revealing that LncDARS‐AS1 was overexpressed in patients who either died (p = 0.0003) or developed metastasis (p = 0.0355) (Figure 1F,G). Compared to patients with lower expression levels, those with higher LncDARS‐AS1 expression exhibited significantly reduced OS (Figure 1H). These findings emphasize the critical role of LncDARS‐AS1 in osteosarcoma metastasis and its potential as a prognostic biomarker, warranting further investigation.

### 3.2 LncDARS‐AS1 Enhances Malignant Proliferation and Metastasis of Osteosarcoma Cells In Vitro and In Vivo

**Figure 2:**
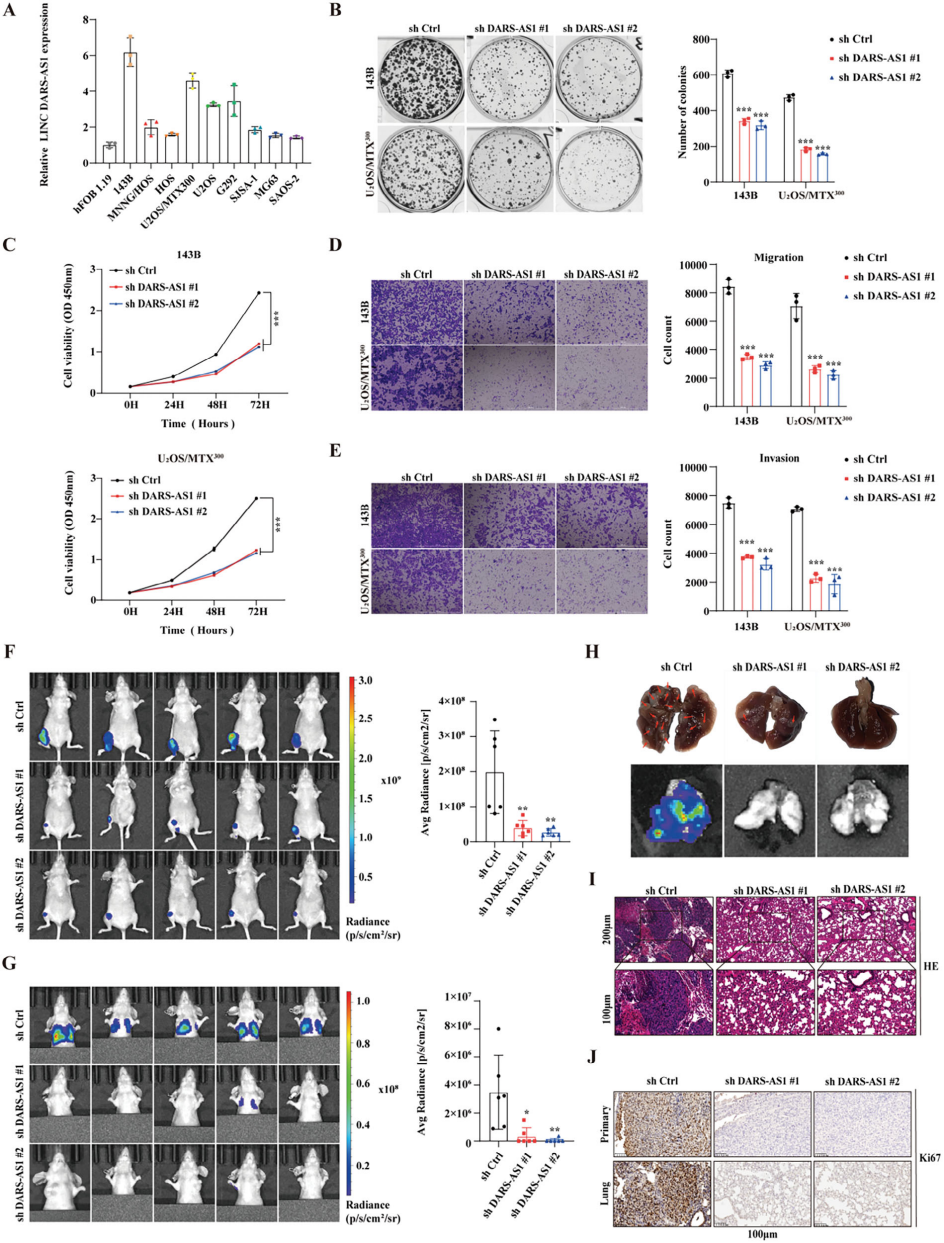
Functional validation of LncDARS‐AS1 in osteosarcoma cells. A) Relative expression levels of LncDARS‐AS1 in nine osteosarcoma cell lines, with markedly higher expression observed in 143B and U2OS/MTX300 compared to the normal osteoblastic cell line Hfob1.19. B) Knockdown of LncDARS‐AS1 significantly reduced the colony formation ability of 143B and U_2_OS/MTX^300^ tumor cells. C) LncDARS‐AS1 knockdown significantly decreased the proliferation of 143B and U_2_OS/MTX^300^ tumor cells. D, E) LncDARS‐AS1 knockdown reduced the migration (D) and invasion (E) abilities of 143B and U_2_OS/MTX^300^ tumor cells. F, G) Representative fluorescent images of tumor formation (F) and lung metastasis (G) in nude mice following LncDARS‐AS1 knockdown. H) Histological examination and fluorescence imaging of lung tissues showed reduced metastatic burden in the LncDARS‐AS1 knockdown group compared to the ShCtrl group. I) Hematoxylin and eosin (H&E) staining of lung tissues showing a reduced number of metastatic lesions in the LncDARS‐AS1 knockdown group compared to the ShCtrl group. J) Immunohistochemistry (IHC) staining for Ki67 showed significantly lower positive staining in the LncDARS‐AS1 knockdown group compared to the control ShCtrl group. ^*^ *P* < 0.05, ^**^ *P* < 0.01, ^***^ *P* < 0.001.

LncDARS‐AS1 expression was assessed across nine osteosarcoma cell lines and the normal osteoblastic cell line Hfob1.19 to examine its differential expression in osteosarcoma. Notably, increased relative expression of LncDARS‐AS1 was observed in the 143B and U2OS/MTX300 tumor cell lines (**Figure** **2**A). Stable knockdown of LncDARS‐AS1 was established in 143B and U_2_OS/MTX^300^ cell lines (Figure S1A, Supporting Information), and stable overexpression was achieved in HOS and U_2_OS cells (Figure S1B, Supporting Information) for subsequent functional assays. LncDARS‐AS1 knockdown significantly impaired colony formation (Figure 2B) and reduced proliferation (Figure 2C) in tumor cells. Migration (Figure 2D) and invasion (Figure 2E) abilities were significantly diminished.

In an orthotopic osteosarcoma model in nude mice, LncDARS‐AS1 knockdown significantly reduced primary tumor growth (Figure 2F) and pulmonary metastasis (Figure 2G) compared with the ShCtrl group. Gross examination and fluorescence imaging of lung tissues (Figure 2H) revealed markedly fewer metastatic lesions in the knockdown group. Histological analysis with hematoxylin and eosin (H&E) staining (Figure 2I) confirmed the reduced metastatic burden. Ki67 immunostaining of both primary and lung lesions (Figure 2J) demonstrated decreased proliferative activity in tumors with LncDARS‐AS1 suppression.

In contrast, overexpression of LncDARS‐AS1 in HOS and U_2_OS tumor cells resulted in significantly enhanced colony formation (Figure S1C, Supporting Information), proliferation (Figure S1D, Supporting Information), migration (Figure S1E, Supporting Information), and invasion (Figure S1F, Supporting Information) abilities in vitro. These findings demonstrate that LncDARS‐AS1 plays a critical role in promoting osteosarcoma cell proliferation and metastasis both in vitro and in vivo.

### 3.3 LncDARS‐AS1 Interacted with ATP1A1 Protein in Osteosarcoma Cells

**Figure 3:**
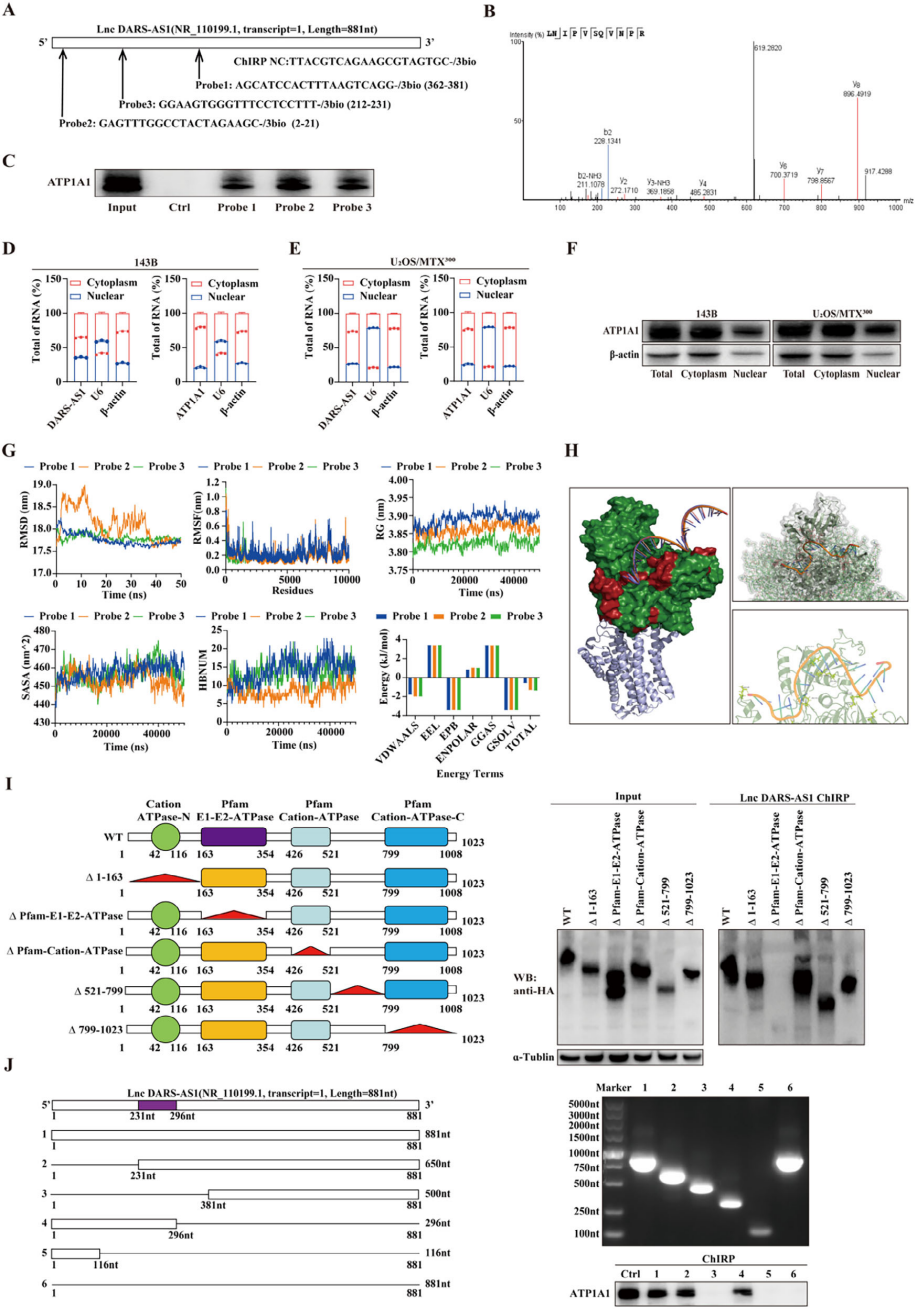
Interaction between LncDARS‐AS1 and ATP1A1 protein in Osteosarcoma Cells. A) Schematic illustration of the biotin‐labeled LncDARS‐AS1 probe used in chromatin isolation by RNA purification (ChIRP) assays. B) Mass spectrometry analysis of proteins retrieved from the ChIRP assay identified ATP1A1 as a specific LncDARS‐AS1 interacting protein. C) Western blot validation of the direct interaction between LncDARS‐AS1 and ATP1A1 identified by ChIRP pull‐down. D–F) Subcellular localization of LncDARS‐AS1 and ATP1A1 in 143B and U2OS/MTX300 osteosarcoma cells, as determined by qPCR and Western blotting, showing predominant cytoplasmic distribution of both molecules. G) Molecular dynamics simulations demonstrate a stable and high‐affinity interaction between LncDARS‐AS1 and ATP1A1. H) Schematic representation of the optimal binding conformation between LncDARS‐AS1 and ATP1A1, highlighting the specificity of the interaction interface. I, J) Schematic representations of truncated constructs of ATP1A1 (I, left) and LncDARS‐AS1 (J, left) used to identify interaction domains. The right panels (I, J) show that the ΔPfam‐E1‐E2‐ATPase domain of ATP1A1 (amino acids 163–354) and the middle region of LncDARS‐AS1 (nucleotides 231–296) are critical for their interaction. These critical binding regions are highlighted in purple.

To elucidate the mechanistic role of LncDARS‐AS1 in osteosarcoma proliferation and metastasis, a comprehensive study was conducted. A specific biotinylated LncDARS‐AS1 nucleic acid probe (**Figure** **3**A) was used for RNA pull‐down (ChIRP), followed by mass spectrometry to identify proteins that interact with LncDARS‐AS1. The probes exhibited high complementarity to the conserved regions of LncDARS‐AS1, ensuring their specificity. Mass spectrometry analysis revealed that ATP1A1 was the most abundant protein co‐precipitated with LncDARS‐AS1 (Figure 3B; Table S2, Supporting Information). To confirm this, the direct interaction between LncDARS‐AS1 and ATP1A1 was validated by Western blot (WB) analysis (Figure 3C). Subcellular localization analysis in 143B and U_2_OS/MTX^300^ osteosarcoma cells, based on qPCR and Western blotting, showed that both LncDARS‐AS1 and ATP1A1 were predominantly localized in the cytoplasm, suggesting that their interaction primarily occurs within this compartment (Figure 3D–F).

To further investigate the molecular interaction, molecular dynamics simulations were applied to analyze the binding between three complementary probes designed to bind to LncDARS‐AS1 and ATP1A1 protein. The results demonstrated that these molecules bind tightly and stably, further supporting their robust interaction (Figure 3G). A schematic representation of their optimal binding pose (Figure 3H) highlighted the stability and specificity of the interaction, emphasizing its crucial role in osteosarcoma cell function and regulation.

To define the regions essential for the interaction, a series of truncated constructs of ATP1A1 and LncDARS‐AS1 were generated based on predicted functional domains (Figure 3I,J, left panels). Domain‐mapping analysis revealed that the ΔPfam‐E1‐E2‐ATPase domain of ATP1A1 (amino acids 163–354) and the central region of LncDARS‐AS1 (nucleotides 231–296) were required for their binding (Figure 3I,J, right panels). These key interaction regions are consistently highlighted in purple in schematic diagrams. These results establish a direct and specific interaction between LncDARS‐AS1 and ATP1A1, highlighting its functional relevance in osteosarcoma and providing mechanistic insight into lncRNA–protein interactions in tumor progression.

### 3.4 Prognostic Significance of ATP1A1 Expression in Osteosarcoma

**Figure 4:**
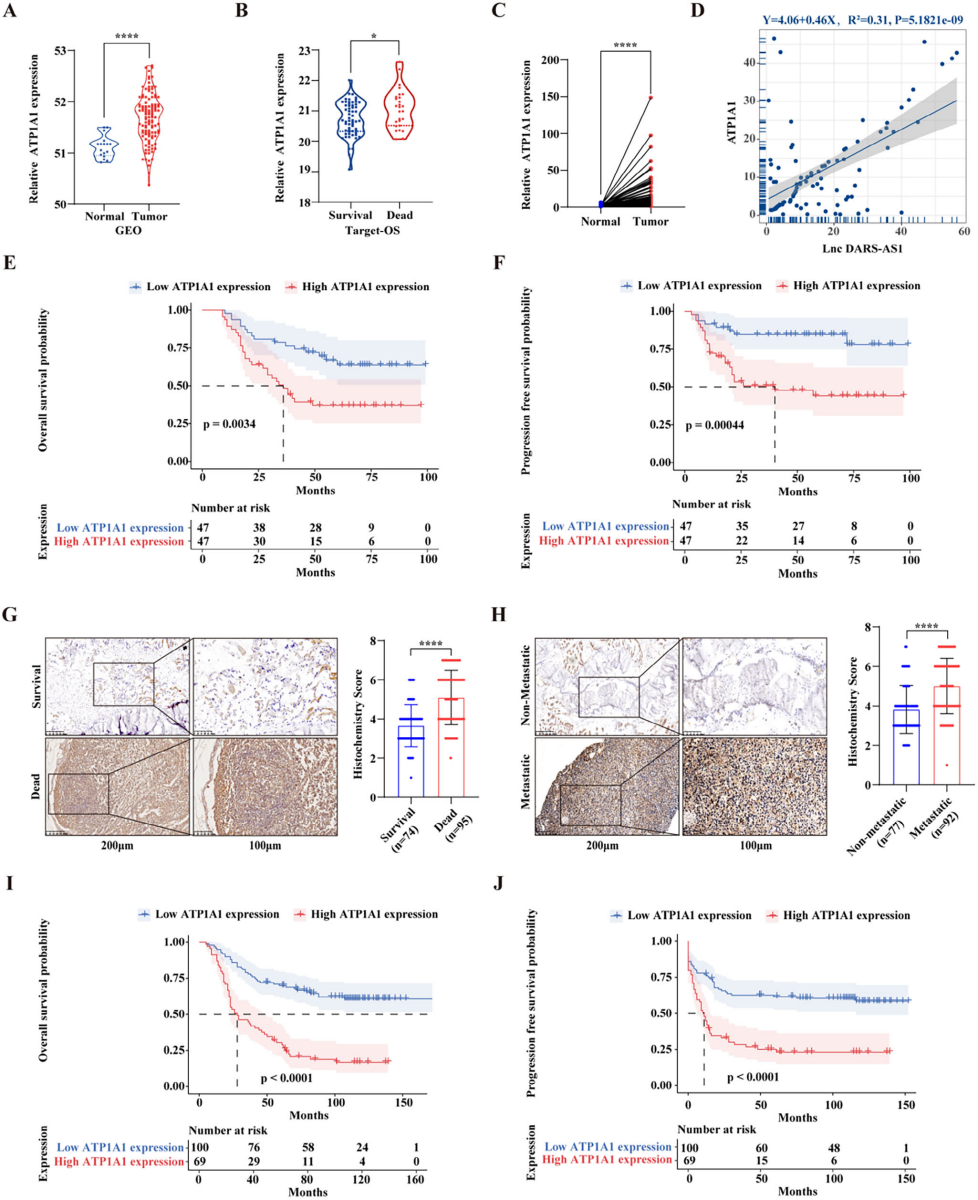
Prognostic significance of ATP1A1 expression in osteosarcoma. A) Integrated analysis of five osteosarcoma‐related gene expression datasets from the GEO database revealed significantly elevated ATP1A1 expression in osteosarcoma tissues compared to normal controls. B) Analysis of the TARGET‐OS dataset showed that ATP1A1 expression was significantly higher in patients who died from osteosarcoma than in those who survived. C) RT–qPCR analysis of paired osteosarcoma and adjacent normal tissues from 94 patients confirmed upregulation of ATP1A1 in osteosarcoma. D) RNA expression levels of LncDARS‐AS1 and ATP1A1 were positively correlated in the same patient cohort. E) In paired RNA samples from 94 osteosarcoma patients, higher ATP1A1 expression was associated with significantly poorer overall survival. F) Progression‐free survival was significantly shorter in the 94 paired RNA samples from patients with higher ATP1A1 expression. G) Immunohistochemical analysis of 169 osteosarcoma tissue samples showed that ATP1A1 positivity was significantly higher in patients who died from the disease. H) ATP1A1 positivity was significantly increased in metastatic osteosarcoma tissues, as determined by immunohistochemical analysis of the same patient cohort. I) In the cohort of 169 osteosarcoma patients, higher ATP1A1 expression assessed by immunohistochemistry was significantly associated with poorer overall survival. J) Higher ATP1A1 expression was significantly associated with worse progression‐free survival in the 169 osteosarcoma patients. ^*^ *p* < 0.05, ^**^ *p* < 0.01, ^***^ *p* < 0.001, and ^****^ *p* < 0.0001.

To investigate the prognostic and functional significance of ATP1A1, the protein that specifically interacts with LncDARS‐AS1 in osteosarcoma, a comprehensive analysis was conducted using five osteosarcoma‐related expression profiling datasets from the GEO database. The combined dataset included 17 normal human cell samples and 114 osteosarcoma samples. The analysis revealed a significant upregulation of ATP1A1 in osteosarcoma tissues (**Figure** **4**A). Further analysis of the TARGET‐OS dataset showed that ATP1A1 expression was significantly higher in patients who succumbed to osteosarcoma compared to those who survived (Figure 4B).

RT‐PCR validation of paired osteosarcoma tissue samples from 94 patients confirmed the upregulation of ATP1A1 in osteosarcoma tissues (Figure 4C), with a positive correlation between the RNA expression levels of LncDARS‐AS1 and ATP1A1 in the same cohort (Figure 4D). Notably, patients with higher ATP1A1 expression exhibited significantly poorer overall survival (Figure 4E) and progression‐free survival (Figure 4F) compared to those with lower ATP1A1 expression.

Among 279 patients initially screened, 100 were excluded due to extensive tumor necrosis, which hindered validation, and 10 were excluded due to missing clinical data. In total, 169 patients were included in the immunohistochemical analysis. Detailed patient information is provided in **Table** **2**. Immunohistochemical analysis revealed significantly higher ATP1A1 positivity in patients who died of disease (Figure 4G) and in those with metastatic osteosarcoma (Figure 4H). Furthermore, patients with high ATP1A1 expression exhibited significantly poorer overall survival (Figure 4I) and progression‐free survival (Figure 4J) compared to those with low expression.

**Table 2:** Clinical and Demographic Characteristics of Patients in IHC Analysis.

Variable	Total
Initial patient	279
Excluded patient	110
Included patient	169
Sex (no. [%])	
Male	113 (66.86%)
Female	56(33.14%)
Age of diagnosis (no.)(years)	
Mean	20.18
Std. dev.	11.19
Median	16
Range	6‐64
Enecking stage (no.[%])	
II	142 (84.02%)
III	27(15.98%)
Relapse (no. [%])	21 (12.43%)
Metastasis(no. [%])	83(49.11%)
Follow‐up time(no.[%])(Months)	
Follow‐up cases	169 (100%)
Mean	68.11
Median	63.00
Range	5‐173
Survival status (no. [%])	
Survival	74(43.79%)
Death	95(56.21%)

### 3.5 Impact of ATP1A1 Expression on Osteosarcoma Cell Functions and Tumor Progression

**Figure 5:**
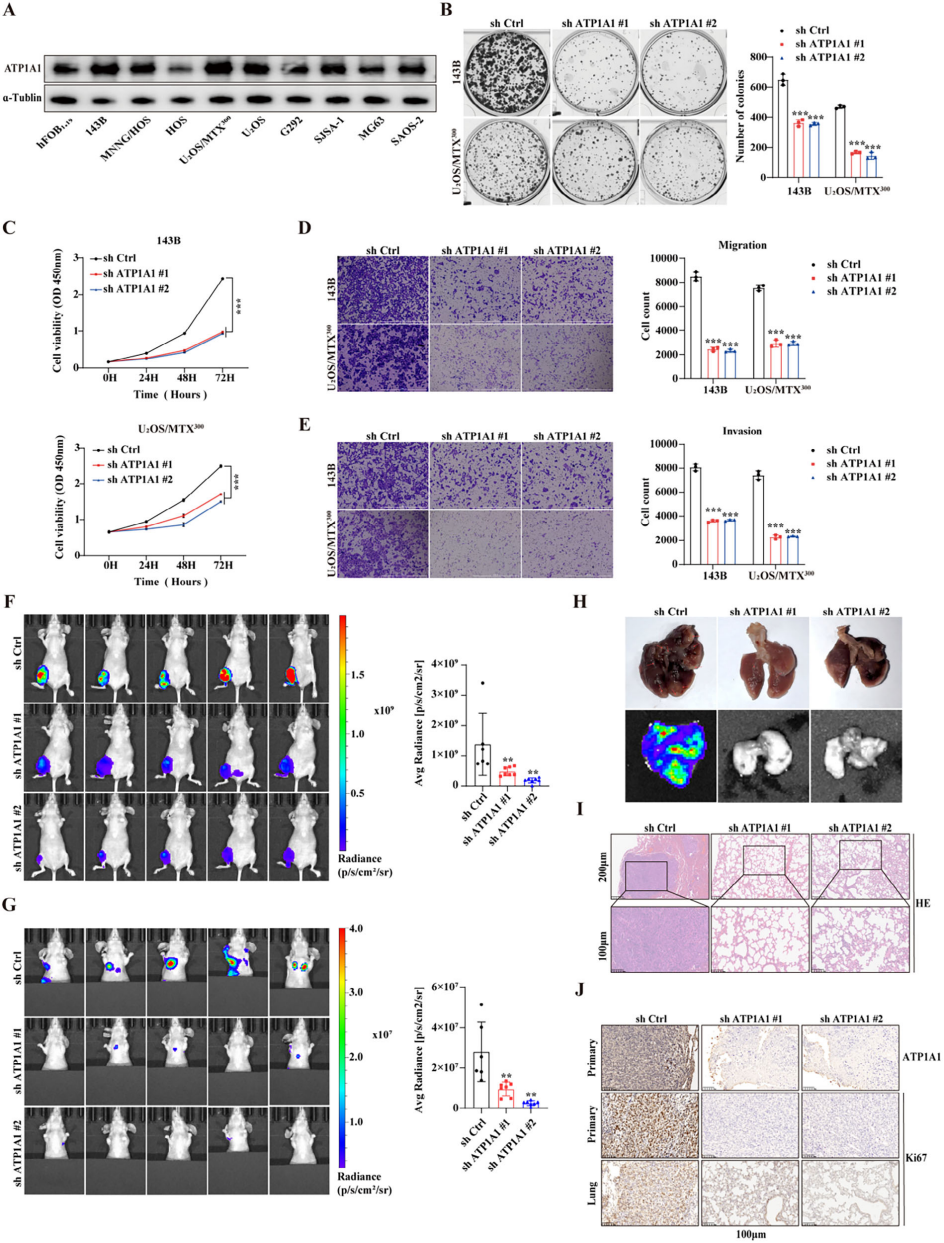
Effects of ATP1A1 expression on osteosarcoma cell functions and tumor progression in vitro and in vivo. A) ATP1A1 expression levels in nine osteosarcoma cell lines and the normal osteoblast cell line Hfob 1.19, showing higher expression in the 143B and U_2_OS/MTX^300^ cell lines. B) Colony formation was significantly reduced in ATP1A1 knockdown 143B and U_2_OS/MTX^300^ cells compared with the control group. C) Proliferation was significantly suppressed in ATP1A1 knockdown 143B and U_2_OS/MTX^300^ cells relative to the control group. D) Transwell migration assays demonstrated significantly impaired migratory capacity in ATP1A1‐knockdown 143B and U_2_OS/MTX^300^ cells. E) Invasion ability was significantly reduced in ATP1A1 knockdown 143B and U_2_OS/MTX^300^ cells. F) In vivo tumorigenicity assays in nude mice revealed a substantial reduction in tumor growth following ATP1A1 knockdown. G) Lung metastatic burden was significantly decreased in the ATP1A1‐knockdown group compared with controls. H) Histological examination and fluorescence imaging of lung tissues revealed a marked reduction in pulmonary metastases in the ATP1A1‐knockdown group. I) Hematoxylin and eosin (H&E) staining of lung sections confirmed a decreased metastatic burden following ATP1A1 knockdown. J) Immunohistochemical staining of ATP1A1 in primary tumors revealed a reduced ATP1A1 positivity rate in primary tumors from the ATP1A1 knockdown group. Ki67 staining demonstrated significantly fewer Ki67‐positive cells in both primary tumors and metastatic lung lesions, indicating reduced proliferative activity. ^*^ *p* < 0.05, ^**^ *p* < 0.01, and ^***^ *p* < 0.001.

To assess the functional impact of ATP1A1 expression in osteosarcoma, cell lines were selected based on ATP1A1 expression levels and compared with the normal osteoblastic cell line Hfob1.19. Screening results identified 143B and U_2_OS/MTX^300^ as exhibiting markedly elevated ATP1A1 expression (**Figure** **5**A). Subsequently, stable ATP1A1 knockdown 143B and U_2_OS/MTX^300^ cell lines were successfully established (Figure S1G, Supporting Information), along with stable ATP1A1 overexpression in HOS and U_2_OS osteosarcoma cell lines (Figure S1H, Supporting Information).

In vitro functional assays demonstrated that ATP1A1 knockdown significantly impaired colony formation (Figure 5B), cell proliferation (Figure 5C), migration (Figure 5D), and invasion (Figure 5E) in the 143B and U_2_OS/MTX^300^ cell lines compared to the control group. In vivo studies further confirmed that ATP1A1 knockdown markedly reduced tumorigenicity (Figure 5F) and lung metastasis (Figure 5G). Gross morphological analysis and fluorescence imaging of lung tissues (Figure 5H) revealed a significant decrease in metastatic lesions in the ATP1A1 knockdown group. Hematoxylin and eosin (HE) staining of lung tissue sections (Figure 5I) further confirmed this reduction.

Immunohistochemical staining revealed reduced ATP1A1 positivity in primary tumors from the ATP1A1 knockdown group. Additionally, Ki67 immunostaining of both primary tumors and lung tissues showed a marked decrease in Ki67‐positive cells following ATP1A1 knockdown (Figure 5J), indicating suppressed tumor cell proliferation.

In contrast, overexpression of ATP1A1 significantly enhanced colony formation (Figure S1I, Supporting Information), cellular proliferation (Figure S1J, Supporting Information), migration (Figure S1K, Supporting Information), and invasion (Figure S1L, Supporting Information) in HOS and U_2_OS cell lines. These findings highlight the critical role of ATP1A1 in osteosarcoma progression. The consistent upregulation of both ATP1A1 and LncDARS‐AS1 suggests a potential regulatory relationship that may contribute to malignancy. Further investigation of this axis may elucidate the molecular mechanisms underlying osteosarcoma development and metastasis.

### 3.6 LncDARS‐AS1 Regulates ATP1A1 Stability via Ubiquitination and the Ubiquitin‐Proteasome Pathway

Previous experiments demonstrated a direct interaction between LncDARS‐AS1 and ATP1A1, with both functioning as oncogenic drivers in osteosarcoma by promoting tumor proliferation and metastasis. Their expression levels are positively correlated in osteosarcoma tissues. To further elucidate the regulatory mechanism underlying this interaction, the effect of modulating LncDARS‐AS1 expression on ATP1A1 levels in osteosarcoma cells was investigated.

**Figure 6:**
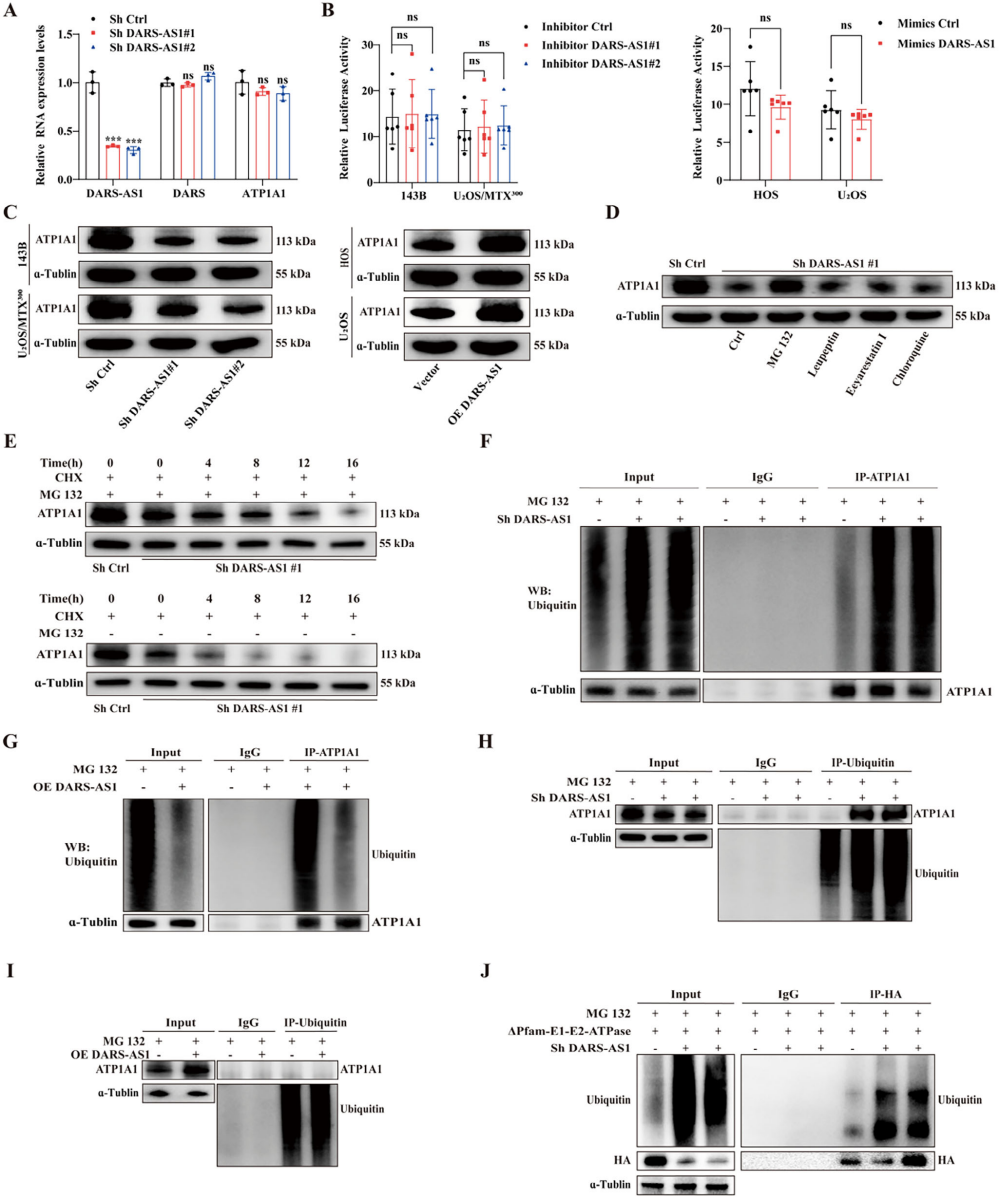
Modulation of LncDARS‐AS1 Affects ATP1A1 Expression, Stability, and Ubiquitination in Osteosarcoma Cells. A) Quantitative PCR analysis of ATP1A1, DARS, and LncDARS‐AS1 expression in LncDARS‐AS1 knockdown and control osteosarcoma cells, showing no significant change in ATP1A1 and DARS mRNA levels. B) Dual‐luciferase reporter assay confirming that modulation of LncDARS‐AS1 does not affect ATP1A1 promoter activity. C) Western blot analysis reveals that LncDARS‐AS1 knockdown reduces ATP1A1 protein levels, while overexpression of LncDARS‐AS1 increases ATP1A1 protein expression. D) Treatment with the proteasome inhibitor MG132 rescues the reduction in ATP1A1 protein levels caused by LncDARS‐AS1 knockdown, indicating that LncDARS‐AS1 modulates ATP1A1 stability via the ubiquitin–proteasome system (UPS). E) Cycloheximide (CHX) chase assay showing that LncDARS‐AS1 knockdown decreases ATP1A1 protein stability, while MG132 treatment significantly extends the half‐life of ATP1A1 protein in LncDARS‐AS1 knockdown cells. F) LncDARS‐AS1 knockdown enhances ATP1A1 ubiquitination and increases the binding of ubiquitin (P4D1) to ATP1A1. G) Overexpression of LncDARS‐AS1 reduces ATP1A1 ubiquitination and decreases P4D1 binding to ATP1A1. H) Co‐immunoprecipitation (co‐IP) using P4D1 antibody shows increased ubiquitin binding to ATP1A1 in LncDARS‐AS1‐knockdown cells. I) Reduced binding of ubiquitin (P4D1) to ATP1A1 in LncDARS‐AS1 overexpression cells. J) Ubiquitination levels of truncated ATP1A1 (Δ163–354aa) are elevated in LncDARS‐AS1 knockdown cells compared with controls, confirming the interaction region critical for degradation regulation.

Quantitative PCR (qPCR) analysis revealed that knockdown of LncDARS‐AS1 had no significant effect on ATP1A1 mRNA levels or the expression of the antisense DARS transcript (**Figure** **6**A), suggesting that LncDARS‐AS1 does not regulate ATP1A1 at the transcriptional level. Consistently, dual‐luciferase reporter assays demonstrated that neither knockdown nor overexpression of LncDARS‐AS1 affected ATP1A1 promoter activity (Figure 6B). In contrast, Western blot analysis revealed that knockdown of LncDARS‐AS1 decreased ATP1A1 protein levels, while overexpression of LncDARS‐AS1 significantly increased ATP1A1 protein abundance (Figure 6C). These findings indicate that LncDARS‐AS1 regulates ATP1A1 expression predominantly at the post‐transcriptional level.

To validate these findings in vivo, RNA was extracted from orthotopic tumor specimens of nude mice in the LncDARS‐AS1 knockdown and control groups. qPCR analysis confirmed a significant reduction in LncDARS‐AS1 expression in the knockdown group, while ATP1A1 mRNA levels showed no significant difference between groups (Figure S1M, Supporting Information). In contrast, immunohistochemical staining revealed markedly reduced ATP1A1 protein expression in the knockdown group compared with the control group (Figure S1N, Supporting Information).

After confirming that LncDARS‐AS1 regulates ATP1A1 expression through specific binding, the involvement of various protein degradation pathways was assessed. 143B cells were treated with the proteasome inhibitor MG132, the lysosomal inhibitor leupeptin, the ER‐associated degradation inhibitor Eeyarestatin I, and the autophagy inhibitor chloroquine. Treatment with MG132 alleviated the decrease in ATP1A1 protein levels induced by LncDARS‐AS1 knockdown (Figure 6D).

Furthermore, Western blot analysis following cycloheximide (CHX) treatment, which inhibits de novo protein synthesis, demonstrated that LncDARS‐AS1 knockdown shortened the half‐life of ATP1A1 protein. Notably, MG132 treatment significantly rescued ATP1A1 protein stability in LncDARS‐AS1‐deficient cells (Figure 6E). These results indicate that LncDARS‐AS1 regulates ATP1A1 stability via the ubiquitin–proteasome system (UPS).

To further validate that LncDARS‐AS1 regulates ATP1A1 ubiquitination through the UPS pathway, ubiquitination levels of ATP1A1 were assessed in osteosarcoma 143B cells with varying LncDARS‐AS1 expression levels following MG132 treatment. Knockdown of LncDARS‐AS1 significantly increased ATP1A1 ubiquitination, and co‐immunoprecipitation (co‐IP) with an ATP1A1 antibody revealed increased ubiquitination marker (P4D1) on ATP1A1 (Figure 6F). Conversely, LncDARS‐AS1 overexpression reduced ATP1A1 ubiquitination, with a corresponding decrease in P4D1 binding to ATP1A1 (Figure 6G). Co‐IP experiments using a ubiquitin antibody (P4D1) further confirmed that LncDARS‐AS1 knockdown enhanced P4D1 binding to ATP1A1 (Figure 6H), while overexpression of LncDARS‐AS1 diminished this interaction (Figure 6I). ATP1A1 (Δ163–354aa) represents a critical site for the interaction between LncDARS‐AS1 and ATP1A1. Comparison of ubiquitination levels of truncated ATP1A1 (Δ163–354aa) between LncDARS‐AS1 knockdown and control groups demonstrated a significant increase in the ubiquitination of ATP1A1 (Δ163–354aa) in the LncDARS‐AS1 knockdown group (Figure 6J).

In summary, these findings demonstrate that LncDARS‐AS1 regulates ATP1A1 protein stability by modulating its ubiquitination. Knockdown of LncDARS‐AS1 enhances ATP1A1 ubiquitination and promotes its proteasomal degradation via the ubiquitin–proteasome system (UPS). This regulation is mediated through direct interaction with the ATP1A1 Δ163–354 amino acid region, highlighting the critical role of LncDARS‐AS1 in maintaining ATP1A1 stability and function. These results underscore the importance of the LncDARS‐AS1–ATP1A1 axis in osteosarcoma cell regulation and malignancy.

### 3.7 LncDARS‐AS1 Inhibits the Ubiquitination Degradation of ATP1A1 Protein by Occupying the Binding Site Between ATP1A1 and UBQLN4

**Figure 7:**
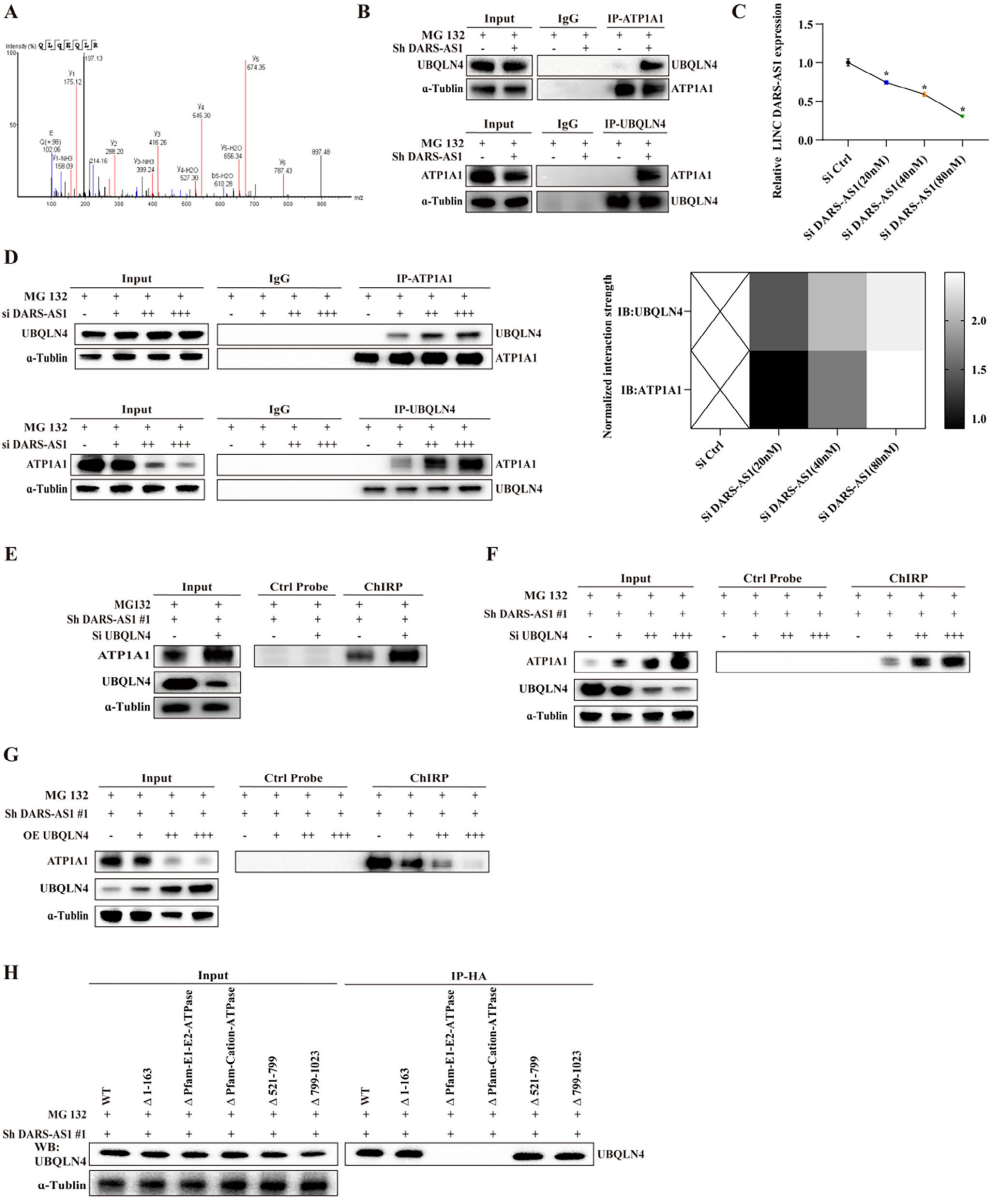
Modulation of UBQLN4 Affects ATP1A1 Stability and Its Interaction with LncDARS‐AS1 in Osteosarcoma Cells. A) Mass spectrometry analysis of co‐immunoprecipitation assays, identifying UBQLN4 as a significantly enriched protein in LncDARS‐AS1 knockdown cells. B) Co‐IP analysis confirmed that ATP1A1 interacts with UBQLN4 specifically in LncDARS‐AS1‐knockdown cells, with no detectable change in total UBQLN4 expression. The upper panel shows Co‐IP using ATP1A1 as the bait, while the lower panel shows Co‐IP using UBQLN4 as the bait. C) Quantitative PCR analysis validating the efficiency of siRNA‐mediated gradient knockdown of LncDARS‐AS1. D) Co‐immunoprecipitation (Co‐IP) analysis confirmed that the interaction between ATP1A1 and UBQLN4 progressively increased in cells with graded knockdown of LncDARS‐AS1. The right panel presents a heatmap illustrating the grayscale intensity trends of the interaction signals under different LncDARS‐AS1 expression levels. The upper heatmap reflects UBQLN4 detected in ATP1A1 immunoprecipitates, while the lower heatmap shows ATP1A1 detected in UBQLN4 immunoprecipitates. Darker shading indicates weaker interaction strength. E) Knockdown of UBQLN4 increases ATP1A1 protein levels in LncDARS‐AS1 knockdown cells, further enhancing the binding between LncDARS‐AS1 and ATP1A1. F) Stepwise knockdown of UBQLN4 in LncDARS‐AS1‐knockdown cells results in progressive elevation of ATP1A1 levels and a corresponding increase in LncDARS‐AS1–ATP1A1 binding. G) Gradual overexpression of UBQLN4 decreases ATP1A1 levels in LncDARS‐AS1 knockdown cells, leading to a reduced interaction between LncDARS‐AS1 and ATP1A1. H) Co‐IP using truncated ATP1A1 mutants reveals that the UBQLN4 binding site overlaps with the LncDARS‐AS1 binding site in the ∆Pfam‐E1‐E2‐ATPase regions.

To further investigate the molecular mechanism by which LncDARS‐AS1 stabilizes ATP1A1 protein in osteosarcoma cells via the ubiquitin‐proteasome system (UPS), co‐immunoprecipitation (co‐IP) followed by mass spectrometry analysis was conducted to compare LncDARS‐AS1‐knockdown cells with control cells. UBQLN4 emerged as a significantly enriched ubiquitin‐related protein, with the highest peptide abundance observed in the LncDARS‐AS1‐knockdown group (**Figure** **7**A; Table S3, Supporting Information). Subsequent co‐IP assays confirmed that ATP1A1 interacted with UBQLN4 specifically in the knockdown group, with no detectable change in overall UBQLN4 expression (Figure 7B). To assess the effect of LncDARS‐AS1 expression on the interaction between ATP1A1 and UBQLN4, osteosarcoma cells with graded LncDARS‐AS1 knockdown were established (Figure 7C). Co‐immunoprecipitation analysis demonstrated that graded knockdown of LncDARS‐AS1 led to a progressive increase in the interaction between ATP1A1 and UBQLN4, as evidenced by both Western blot signals and the corresponding grayscale intensity heatmap (Figure 7D).

To elucidate the role of UBQLN4 in LncDARS‐AS1–mediated stabilization of ATP1A1, the effects of UBQLN4 knockdown on ATP1A1 protein levels were examined. Chromatin isolation by RNA purification (ChIRP) assays was employed to assess the interaction between LncDARS‐AS1 and ATP1A1. In 143B cells with reduced LncDARS‐AS1 expression, UBQLN4 knockdown significantly increased ATP1A1 protein levels and enhanced the LncDARS‐AS1–ATP1A1 interaction (Figure 7E). To further investigate the influence of UBQLN4 expression on this interaction, 143B cells with graded UBQLN4 knockdown were generated. Stepwise reduction of UBQLN4 expression in LncDARS‐AS1‐knockdown cells led to a progressive increase in ATP1A1 protein levels and enhanced binding between ATP1A1 and UBQLN4 (Figure 7F). Conversely, elevated UBQLN4 expression markedly decreased ATP1A1 protein levels and reduced the ATP1A1–UBQLN4 interaction in LncDARS‐AS1‐deficient cells (Figure 7G). These findings suggest that LncDARS‐AS1 may stabilize ATP1A1 by competitively occupying the UBQLN4‐binding region on ATP1A1, thereby preventing its ubiquitination and proteasomal degradation.

The ∆Pfam‐E1‐E2‐ATPase domain (aa 163–354) was previously identified as the specific binding region of ATP1A1 for LncDARS‐AS1. To determine whether LncDARS‐AS1 prevents UBQLN4 from binding to ATP1A1 by occupying this region, a series of truncated ATP1A1 mutant constructs were transfected into LncDARS‐AS1‐knockdown 143B cells. Co‐immunoprecipitation assays revealed that UBQLN4 interacts with ATP1A1 through a region spanning the ∆Pfam‐E1‐E2‐ATPase (aa 163–354) and ∆Pfam‐Cation‐ATPase (aa 426–521) domains, partially overlapping with the LncDARS‐AS1 binding site (Figure 7H).

Collectively, these findings delineate a mechanism by which LncDARS‐AS1 preserves ATP1A1 protein stability through competitive occupation of the UBQLN4‐binding interface on ATP1A1, thereby inhibiting its ubiquitination and degradation.

### 3.8 Rescue Experiment Reveals the Functional Role of ATP1A1 in LncDARS‐AS1 Mediated Osteosarcoma Malignancy

**Figure 8:**
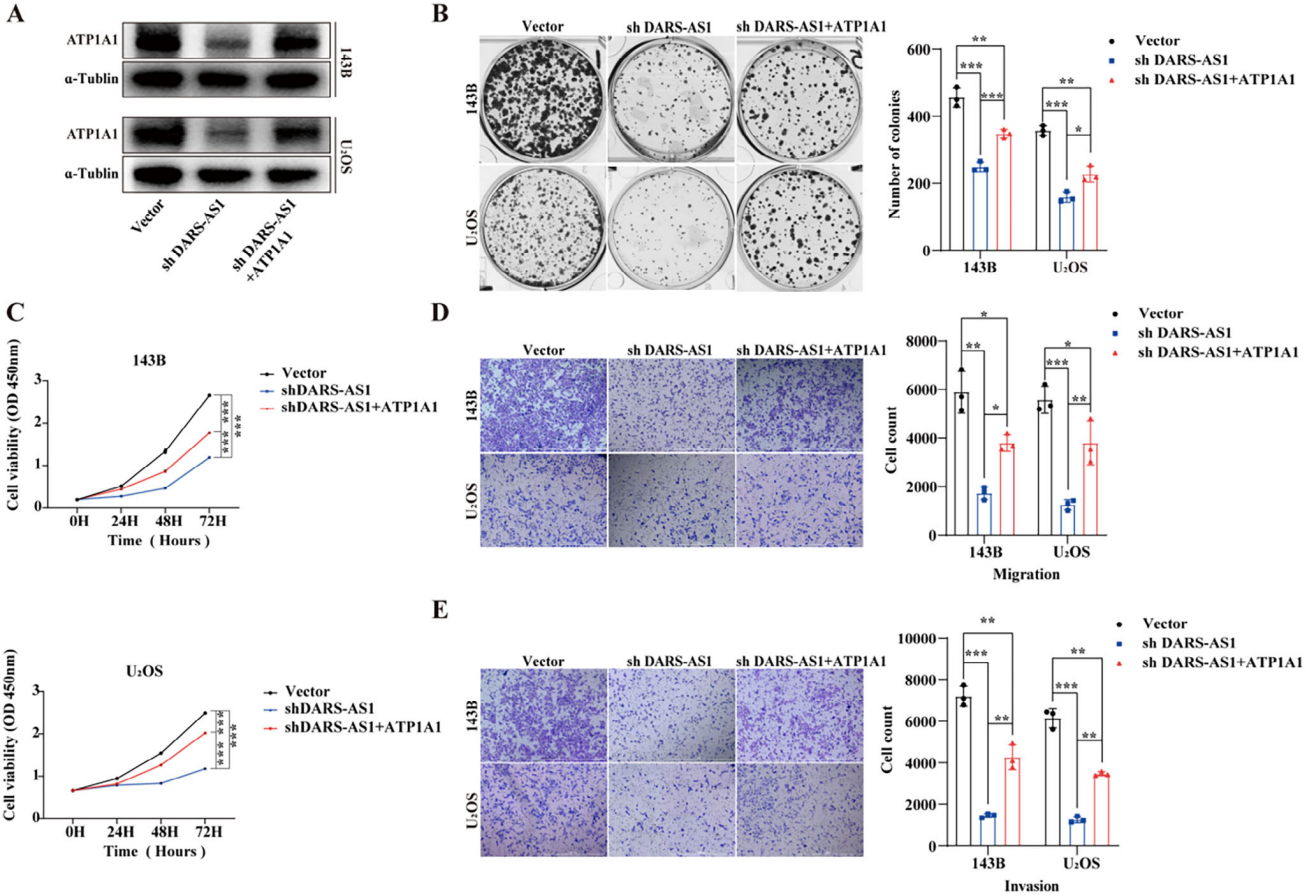
Rescue Experiments Assessing the Role of ATP1A1 in LncDARS‐AS1‐Mediated Osteosarcoma Cell Proliferation and Metastasis. A) LncDARS‐AS1 was stably knocked down in 143B and U_2_OS cells, followed by ATP1A1 overexpression to assess functional compensation. B) Colony formation assay showing that ATP1A1 overexpression partially rescues the inhibitory effect of LncDARS‐AS1 knockdown on cell growth. C) Proliferation assay indicating recovery of cell proliferation upon ATP1A1 overexpression. D) Migration assay demonstrating the reversal of LncDARS‐AS1 knockdown‐induced migratory suppression by ATP1A1 overexpression. E) Invasion assay confirming restoration of invasion ability following ATP1A1 overexpression in LncDARS‐AS1 knockdown cells. ^*^ *p* < 0.05, ^**^ *p* < 0.01 and ^***^ *p* < 0.001.

Initial findings established that both LncDARS‐AS1 and ATP1A1 are markedly overexpressed in osteosarcoma and act as oncogenic drivers by promoting tumor cell proliferation and metastasis. Mechanistically, LncDARS‐AS1 stabilizes ATP1A1 protein expression by occupying the UBQLN4‐binding interface on ATP1A1, thereby preventing its ubiquitination and subsequent proteasomal degradation. To determine whether the oncogenic effects of LncDARS‐AS1 are mediated via ATP1A1, rescue experiments were conducted in the 143B and U_2_OS osteosarcoma cell lines. LncDARS‐AS1 was stably knocked down, followed by ATP1A1 overexpression, enabling the assessment of the functional interaction between these molecules in regulating tumor growth and metastasis (**Figure** **8**A). Colony formation (Figure 8B), proliferation (Figure 8C), migration (Figure 8D), and invasion (Figure 8E) assays revealed that ATP1A1 overexpression partially alleviated the inhibitory effects of LncDARS‐AS1 knockdown on cell proliferation and metastatic potential. These results underscore the pivotal role of ATP1A1 in LncDARS‐AS1‐mediated oncogenesis.

These findings identify ATP1A1 as a key downstream effector in the LncDARS‐AS1 oncogenic pathway and further highlight its contribution to tumor cell aggressiveness. To deepen mechanistic understanding, subsequent analyses will assess Na⁺/K⁺‐ATPase (NKA) enzymatic activity, a core function of ATP1A1, to explore its involvement in promoting osteosarcoma progression and metastasis.

### 3.9 LncDARS‐AS1 and ATP1A1 Expression Regulate the Activity of Na⁺/K⁺‐ATPase

Na⁺/K⁺‐ATPase (NKA) is a membrane‐bound enzyme complex essential for maintaining intracellular ionic homeostasis by actively transporting sodium and potassium ions across the plasma membrane, thereby regulating membrane potential and numerous cellular processes. ATP1A1, the catalytic α1 subunit of Na^+^/K^+^ ATPase (NKA), is indispensable for its enzymatic activity. Perturbations in ATP1A1 expression or stability can compromise NKA function and contribute to pathological alterations. Previous work demonstrated that LncDARS‐AS1 binds specifically to ATP1A1 and prevents its ubiquitination‐mediated degradation by occupying the UBQLN4 binding interface.

**Figure 9:**
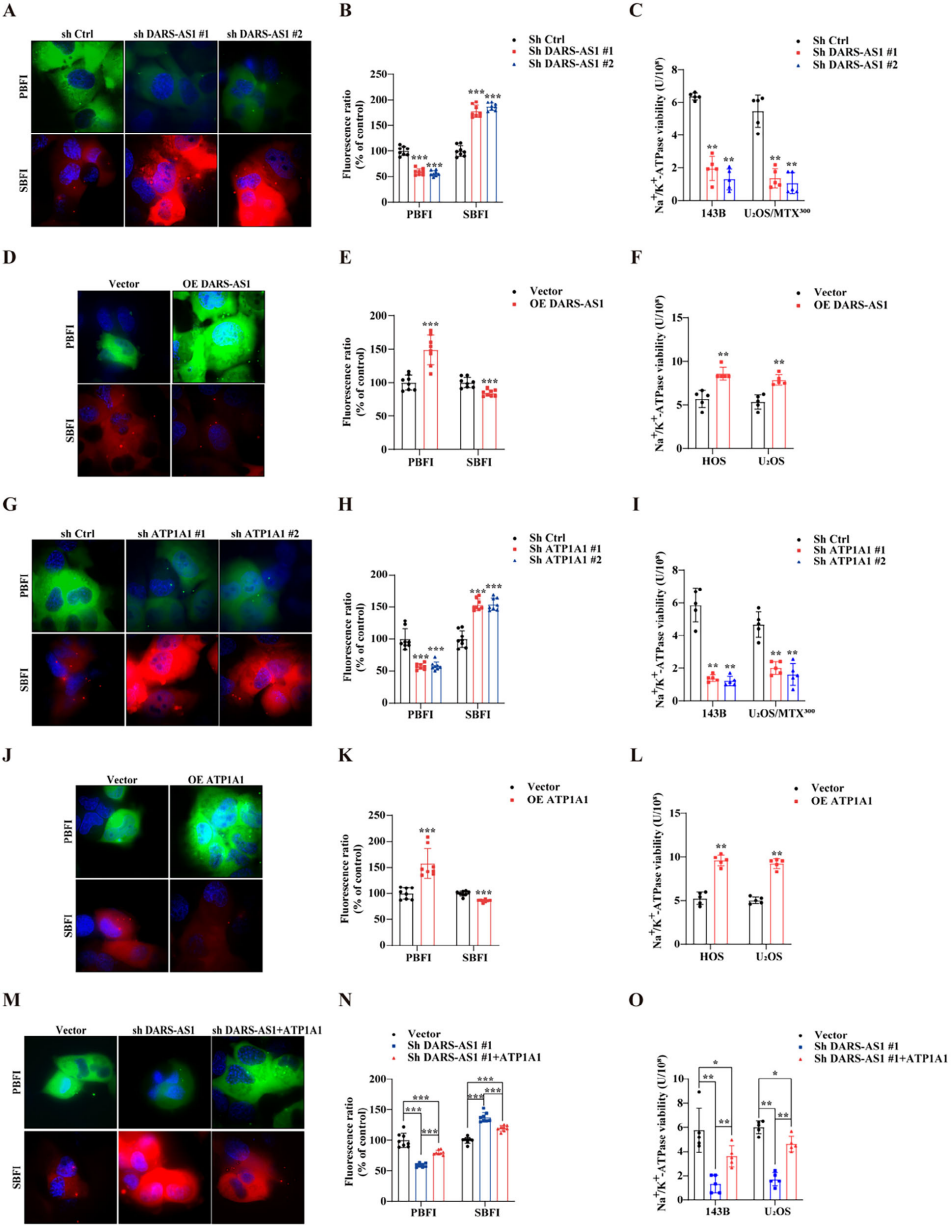
Impact of LncDARS‐AS1 and ATP1A1 expression on ion concentration and Na⁺/K⁺‐ATPase activity in osteosarcoma cells. A) Super‐resolution microscopy showing reduced potassium and increased sodium ion fluorescence intensities in LncDARS‐AS1 knockdown osteosarcoma cells. B) Quantitative analysis of intracellular ion concentrations confirms a significant decrease in potassium and an increase in sodium levels following LncDARS‐AS1 knockdown. C) Na⁺/K⁺‐ATPase activity assay reveals a marked reduction in enzymatic activity in cells with LncDARS‐AS1 knockdown. D) Super‐resolution microscopy of LncDARS‐AS1 overexpressing cells shows elevated potassium and reduced sodium ion fluorescence intensities. E) Quantification of intracellular ion concentrations indicates a significant increase in potassium and a decrease in sodium levels upon LncDARS‐AS1 overexpression. F) Na⁺/K⁺‐ATPase activity assay showing enhanced Na⁺/K⁺‐ATPase activity following LncDARS‐AS1 overexpression. G) Super‐resolution imaging of ATP1A1 knockdown cells demonstrates reduced potassium and increased sodium ion intensities. H) Quantitative ion analysis confirms decreased potassium and increased sodium concentrations following ATP1A1 knockdown. I) ATP1A1 knockdown leads to a significant reduction in Na⁺/K⁺‐ATPase activity. J) Super‐resolution microscopy of ATP1A1 overexpressing cells shows elevated potassium and reduced sodium ion fluorescence intensities. K) Quantification of intracellular ion concentrations indicates a significant increase in potassium and a decrease in sodium levels upon ATP1A1 overexpression. L) Overexpression of ATP1A1 significantly enhances Na⁺/K⁺‐ATPase activity. M) In LncDARS‐AS1‐knockdown cells, ATP1A1 overexpression reverses the reduction in potassium and the increase in sodium ion fluorescence intensities. N) Quantification ion analysis confirms that ATP1A1 overexpression rescues potassium depletion and sodium accumulation induced by LncDARS‐AS1 knockdown. O) ATP1A1 overexpression partially restores Na⁺/K⁺‐ATPase activity in LncDARS‐AS1‐deficient cells. ^*^ *p* < 0.05, ^**^ *p* < 0.01, and ^***^ *p* < 0.001.

To evaluate the functional consequences of this interaction, intracellular ion concentrations were assessed using potassium‐binding (PBFI) and sodium‐binding (SBFI) fluorescent indicators. Super‐resolution microscopy revealed reduced potassium and elevated sodium signals in LncDARS‐AS1 knockdown 143B cells (**Figure** **9**A), findings corroborated by quantitative fluorescence measurements using a microplate reader (Figure 9B). Consistent with these ionic imbalances, Na⁺/K⁺‐ATPase activity was significantly suppressed in the LncDARS‐AS1 knockdown group (Figure 9C). Conversely, overexpression of LncDARS‐AS1 restored potassium levels, reduced intracellular sodium, and enhanced Na^+^/K^+^ ATPase (NKA) activity (Figure 9D–F).

Parallel experiments manipulating ATP1A1 expression yielded comparable results. ATP1A1 knockdown decreased potassium levels, increased sodium concentrations (Figure 9G,H), and impaired NKA activity (Figure 9I), whereas ATP1A1 overexpression restored ionic homeostasis and NKA function (Figure 9J–L). These findings indicate that changes in the expression levels of LncDARS‐AS1 and ATP1A1 significantly impact NKA function and activity.

Notably, ATP1A1 overexpression in LncDARS‐AS1‐knockdown cells not only rescued intracellular ion imbalance but also partially restored NKA activity (Figure 9M–O). These findings confirm ATP1A1 as a functional effector of LncDARS‐AS1 and establish a mechanistic link between LncDARS‐AS1 expression, ATP1A1 stabilization, and NKA‐mediated ion regulation.

In summary, LncDARS‐AS1 modulates NKA activity by stabilizing ATP1A1, thereby maintaining ionic equilibrium in osteosarcoma cells. This novel regulatory axis underscores the pathological relevance of LncDARS‐AS1 in osteosarcoma progression and highlights its potential as a therapeutic target for restoring ion homeostasis and mitigating tumor aggressiveness.

### 3.10 Digoxin Inhibits Osteosarcoma Proliferation and Metastasis via Na⁺/K⁺‐ATPase Suppression

**Figure 10:**
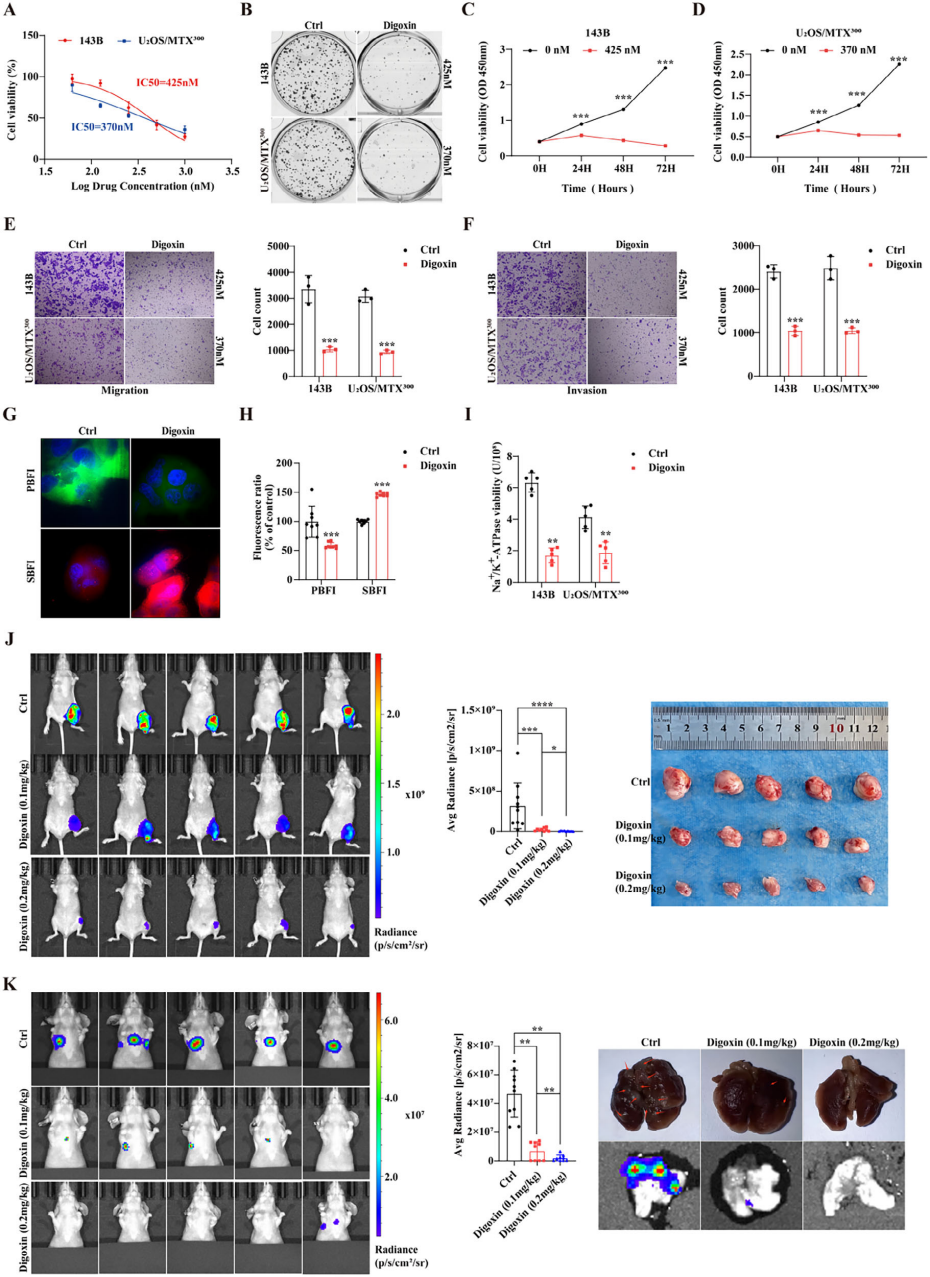
Digoxin suppresses osteosarcoma cell proliferation, migration, invasion, and Na⁺/K⁺‐ATPase (NKA) activity in vitro and in vivo. A) IC₅₀ determination for digoxin in osteosarcoma cell lines 143B and U_2_OS/MTX^300^ identified inhibitory concentrations of 425 and 370 nm, respectively. B) Representative colony formation assay images showing a marked reduction in colony number following digoxin treatment. C, D) CCK‐8 assays quantifying decreased cell proliferation in 143B (C) and U2OS/MTX300 (D) cells after digoxin exposure. E) Migration assay demonstrating that digoxin treatment significantly inhibits osteosarcoma cell migration. F) Invasion assay showing that digoxin treatment markedly inhibits invasive capacity in osteosarcoma cells. G) Super‐resolution microscopy imaging reveals significant differences in the fluorescence intensity of potassium (K⁺) and sodium (Na⁺) ion probes in osteosarcoma cells after digoxin treatment, indicating a decrease in K⁺ and an increase in Na⁺ concentration compared to untreated cells. H) Quantitative analysis confirms a significant decrease in intracellular K⁺ and an increase in Na⁺ concentrations after digoxin treatment. I) Na⁺/K⁺‐ATPase activity assay demonstrating substantial enzymatic inhibition in response to digoxin treatment. J) In vivo assessment of tumor growth inhibition in osteosarcoma‐bearing mice following treatment with 0.1 or 0.2 mg kg^−1^ digoxin. Tumor growth was significantly reduced in both treatment groups, with a more pronounced inhibition observed in the 0.2 mg kg^−1^ group compared to the 0.1 mg kg^−1^ group. K) In vivo evaluation of lung metastasis shows that digoxin significantly suppresses metastatic burden in a dose‐dependent manner, with greater reduction observed at the 0.2 mg kg^−1^ dose. *p* < 0.05, ^**^ *p* < 0.01, and ^***^ *p* < 0.001.

This study establishes that LncDARS‐AS1 promotes osteosarcoma progression by regulating ATP1A1 expression and modulating Na⁺/K⁺‐ATPase (NKA) activity. Given that digoxin is a well‐characterized NKA inhibitor, its therapeutic potential was further explored in the context of osteosarcoma. The half‐maximal inhibitory concentration (IC₅₀) of digoxin was determined to be 425 nm for 143B cells and 370 nm for U2OS/MTX300 cells (**Figure** **10**A). At these IC50 concentrations, digoxin significantly inhibited colony formation, cell proliferation, migration, and invasion in osteosarcoma cells (Figure 10B–F).

To assess the functional impact of digoxin on NKA activity, fluorescent ion indicators revealed decreased potassium and increased sodium ion signals following treatment (Figure 10G). These findings were quantitatively confirmed by microplate‐based assays, which showed a significant reduction in intracellular potassium and an increase in sodium levels (Figure 10H). Concordantly, NKA activity was markedly suppressed in digoxin‐treated cells (Figure 10I). In vivo, mice bearing orthotopic osteosarcoma tumors were randomized into control, 0.1 and 0.2 mg kg^−1^ digoxin treatment groups. Tumor growth and lung metastasis were significantly suppressed in a dose‐dependent manner, with the 0.2 mg kg^−1^ group exhibiting the greatest therapeutic effect (Figure 10J,K).

Collectively, these results demonstrate that digoxin mimics the effects of LncDARS‐AS1 knockdown by inhibiting NKA function, leading to disrupted ion homeostasis and impaired tumor cell viability. The convergence of pharmacological (digoxin) and molecular (LncDARS‐AS1) inhibition on NKA activity underscores a potential therapeutic avenue in osteosarcoma through modulation of membrane ion transport.

## 4 Discussion

Increasing evidence has underscored the pivotal role of long non‐coding RNAs (lncRNAs) in the initiation and progression of various cancers.^[^ ^23^, ^24^, ^25^, ^26^ ^]^ In particular, the dysregulation of specific lncRNAs has been strongly linked to osteosarcoma development, suggesting their potential involvement in multiple stages of tumorigenesis.^[^ ^9^, ^27^, ^28^ ^]^ For example, the DARS‐AS1/RBM39 axis has emerged as a potential therapeutic target in multiple myeloma.^[^ ^10^ ^]^ Additionally, LncDARS‐AS1 has been implicated in promoting the progression of clear cell renal cell carcinoma through the upregulation of DARS.^[^ ^12^ ^]^ Furthermore, overexpression of LncDARS‐AS1 significantly enhances the migration and invasion of triple‐negative breast cancer (TNBC) cells by activating the NF‐κB/STAT3 signaling pathway.^[^ ^13^ ^]^

In this study, the expression profile of LncDARS‐AS1 in osteosarcoma was investigated by integrating non‐coding RNA microarray data from three paired patient samples, whole transcriptome sequencing (WTS) data from 31 patients, and RNA sequencing data from a previously published cohort.^[^ ^14^ ^]^ Elevated LncDARS‐AS1 expression was consistently observed in osteosarcoma patients with pulmonary metastases. Subsequent analysis of RNA samples from 217 clinical specimens revealed that high LncDARS‐AS1 expression was significantly associated with reduced overall survival (OS) and progression‐free survival (PFS). Independent validation using the TARGET‐OS dataset further confirmed that LncDARS‐AS1 expression was elevated in patients who developed lung metastases or succumbed to the disease and was negatively correlated with OS. These findings highlight the potential clinical relevance of LncDARS‐AS1 as a prognostic biomarker in osteosarcoma, particularly in metastatic progression. The primary objective of this study was to elucidate the biological function of LncDARS‐AS1 and to define its mechanistic role in regulating osteosarcoma metastasis.

Recent studies have reported overexpression of LncDARS‐AS1 in triple‐negative breast cancer (TNBC), where its silencing sensitizes tumor cells to doxorubicin (DOX) by suppressing TGF‐β/Smad3 pathway–induced autophagy, thereby enhancing anti‐tumor efficacy.^[^ ^13^ ^]^ Additionally, the large‐scale CRISPRi screening of 971 cancer‐related lncRNAs has identified LncDARS‐AS1 as being significantly associated with malignant phenotypes in tumor cells.^[^ ^11^ ^]^

In the present study, comprehensive in vitro and in vivo functional assays demonstrated that downregulation of LncDARS‐AS1 significantly suppressed the proliferation and metastatic potential of osteosarcoma cells. Given that lncRNA functions are often dependent on subcellular localization^[^ ^29^ ^]^, subcellular fractionation and RNA pull‐down assays revealed that LncDARS‐AS1 predominantly resides in the cytoplasm and specifically interacts with ATP1A1. Mass spectrometry identified ATP1A1 as the principal LncDARS‐AS1 binding protein, and this interaction was further supported by molecular docking and dynamics simulations, which indicated a stable and specific binding interface. Moreover, constructing truncated versions of both ATP1A1 and LncDARS‐AS1 revealed that the interaction between the ∆Pfam‐E1‐E2‐ATPase domain of ATP1A1 (amino acids 163–354) and the central region of LncDARS‐AS1 (nucleotides 231–296) is essential for their binding. Collectively, these findings suggest that the cytoplasmic interaction between LncDARS‐AS1 and ATP1A1 plays a pivotal role in regulating the oncogenic behavior of osteosarcoma cells.

Previous studies have established that long non‐coding RNAs (lncRNAs) can directly interact with downstream target proteins, modulating their ubiquitination levels and thereby influencing tumor progression.^[^ ^30^, ^31^, ^32^, ^33^ ^]^ In the present study, the regulatory effect of LncDARS‐AS1 on ATP1A1 was examined using dual‐luciferase reporter assays and Western blotting. The findings reveal that knockdown of LncDARS‐AS1 does not affect ATP1A1 mRNA expression but leads to a significant reduction in ATP1A1 protein levels, accompanied by an increase in its ubiquitination.

Notably, treatment with the proteasome inhibitor MG132 alleviated the decrease in ATP1A1 protein expression, indicating the involvement of the ubiquitin‐proteasome system (UPS) in this regulatory mechanism. Conversely, overexpression of LncDARS‐AS1 elevated ATP1A1 protein abundance and reduced its ubiquitination. These results suggest that LncDARS‐AS1 modulates ATP1A1 protein stability by regulating its ubiquitination, a process primarily mediated by the ubiquitin‐proteasome system (UPS). Further co‐immunoprecipitation (co‐IP) assays combined with mass spectrometry analysis identified UBQLN4 as a key modulator in the LncDARS‐AS1‐mediated regulation of ATP1A1 ubiquitination. UBQLN4, also known as A1UP, is a UBL‐UBA protein that contains both ubiquitin‐like (UBL) and ubiquitin‐associated (UBA) domains, which facilitate its binding to ubiquitinated proteins and interaction with the proteasome. UBL‐UBA proteins are known to regulate protein degradation through their interaction with various substrates.^[^ ^34^, ^35^ ^]^ Mechanistically, LncDARS‐AS1 was found to bind the ∆Pfam‐E1‐E2‐ATPase domain of ATP1A1, a region essential for Na⁺/K⁺‐ATPase activity, and compete with UBQLN4 for access to this domain. By inhibiting UBQLN4 binding, LncDARS‐AS1 enhances its interaction with ATP1A1, thereby stabilizing ATP1A1 protein levels and enhancing its functional activity. This post‐transcriptional stabilization ultimately promotes osteosarcoma cell proliferation and metastatic potential.

ATP1A1, a critical subunit of the Na^+^/K^+^ ATPase (NKA), is responsible for ATP hydrolysis and the exchange of sodium and potassium ions across the cell membrane.^[^ ^36^, ^37^, ^38^ ^]^ The proper functioning of ATP1A1 is indispensable for maintaining cellular ion homeostasis and ensuring neuronal physiological activities.^[^ ^37^ ^]^ Mutations in ATP1A1 lead to the dysfunction of NKA, resulting in abnormal cation permeability, membrane depolarization, and exacerbation of physiological pump activity loss, which in turn may trigger various diseases.^[^ ^39^ ^]^ Moreover, overexpression of ATP1A1 has been strongly linked to the malignant progression of multiple cancers, including non‐small cell lung cancer^[^ ^40^ ^]^ and pancreatic ductal adenocarcinoma (PDAC).^[^ ^41^ ^]^ Modulation in the function of NKA or the expression of its subunits significantly affects cell adhesion, migration, and motility.^[^ ^42^, ^43^ ^]^ Notably, previous studies have suggested that overexpression of ATP1A1 in cancer cells and fibroblasts promotes epithelial‐mesenchymal transition (EMT), which enhances tumor invasiveness and metastasis.^[^ ^41^ ^]^ Additionally, ATP1A1 deficiency in T cells leads to increased reactive oxygen species (ROS) accumulation and reduced intracellular potassium levels, resulting in T cell exhaustion and further promoting tumor progression.^[^ ^44^ ^]^

Analysis of high‐throughput sequencing data from osteosarcoma patients in the GEO and TARGET‐OS datasets revealed significantly elevated ATP1A1 expression in tumor tissues, particularly among patients with poor clinical outcomes. Further analysis of paired human osteosarcoma tissues confirmed the upregulation of ATP1A1 mRNA, with its high expression strongly associated with poor prognosis. Immunohistochemical analysis of 169 tumor samples further supported the link between high ATP1A1 expression and adverse prognosis in osteosarcoma patients. Functional assays demonstrated that knockdown of ATP1A1 significantly suppressed osteosarcoma cell proliferation and metastasis. Moreover, alterations in the expression of either LncDARS‐AS1 or ATP1A1 impacted Na^+^/K^+^ ATPase (NKA) function and activity. Rescue experiments demonstrated that overexpressing ATP1A1 in LncDARS‐AS1 knockdown cells effectively countered the inhibition of cell proliferation and metastasis and partially restored the NKA function that had been suppressed due to reduced LncDARS‐AS1 expression. These findings indicate that LncDARS‐AS1 regulates NKA activity by modulating ATP1A1 protein stability, underscoring the functional interplay between these two molecules in osteosarcoma progression. This regulatory axis constitutes a critical component of the tumor's molecular machinery, with direct implications for cellular proliferation, invasion, and ion homeostasis. Collectively, the results provide novel insights into the post‐transcriptional regulation of ATP1A1 by LncDARS‐AS1 and identify both molecules as promising candidates for therapeutic intervention and prognostic stratification in osteosarcoma.

Na/K‐ATPase (NKA) is a pivotal membrane‐bound enzyme responsible for maintaining cellular ion balance through the active transport of sodium and potassium ions across the plasma membrane. Disruption of NKA function has been implicated in the pathogenesis and progression of several malignancies. Cardiac glycosides, a class of naturally occurring compounds, are well known for their ability to specifically bind to and inhibit NKA. Tumor cells exhibit heightened sensitivity to these compounds, highlighting their potential as therapeutic agents in cancer treatment.^[^ ^45^, ^46^, ^47^ ^]^ Digoxin, a well‐established NKA‐specific inhibitor, has been widely used in clinical settings for the treatment of heart failure and arrhythmias.^[^ ^48^ ^]^ Increasing evidence suggests that digoxin also possesses significant anti‐cancer properties, inhibiting tumor cell proliferation and metastasis, thereby positioning it as a promising candidate for cancer therapy.^[^ ^45^, ^49^, ^50^ ^]^ In this study, it was confirmed through both in vitro and in vivo experiments that digoxin significantly inhibits osteosarcoma cell proliferation and metastasis. Additionally, digoxin markedly suppresses the function and activity of Na/K‐ATPase (NKA), consistent with its known NKA‐inhibiting properties.

This study delineates the critical roles of the oncogenic long non‐coding RNA LncDARS‐AS1 and the Na⁺/K⁺‐ATPase subunit ATP1A1 in driving pulmonary metastasis in osteosarcoma. Functional assays revealed that LncDARS‐AS1 directly binds to ATP1A1, competitively inhibiting its interaction with the ubiquitin‐binding protein UBQLN4. This inhibition prevents ubiquitin‐mediated proteasomal degradation of ATP1A1, thereby stabilizing the protein and enhancing Na⁺/K⁺‐ATPase (NKA) function. The resulting increase in NKA activity disrupts intracellular ion homeostasis and promotes tumor cell proliferation and metastatic dissemination, uncovering a previously uncharacterized post‐transcriptional regulatory pathway in osteosarcoma progression. However, this regulatory mechanism has so far been identified only in osteosarcoma and has not yet been validated in other malignancies. A broader pan‐cancer analysis of LncDARS‐AS1 expression and function would help determine whether this axis is unique to osteosarcoma or represents a more generalizable cancer vulnerability. Such analysis could shed light on whether LncDARS‐AS1‐mediated regulation of ATP1A1 and NKA activity might serve as a target in other cancer types, thereby offering a potential therapeutic avenue for a wider range of malignancies.

Importantly, these findings highlight the translational relevance of the LncDARS‐AS1‐ATP1A1 axis for therapeutic intervention. Digoxin administered at clinically relevant doses effectively inhibits osteosarcoma cell growth and metastasis. These results provide strong evidence supporting the potential therapeutic application of digoxin in osteosarcoma. Nevertheless, clinical application of digoxin remains limited by its narrow therapeutic index, potential cardiotoxicity, and off‐target effects. These challenges necessitate rigorous preclinical evaluation, particularly in the context of long‐term administration and combinatorial strategies with standard chemotherapeutic regimens. Despite these limitations, the study reveals a previously unrecognized post‐transcriptional regulatory mechanism by which LncDARS‐AS1 stabilizes ATP1A1 and enhances Na⁺/K⁺‐ATPase (NKA) activity, ultimately promoting osteosarcoma progression. This axis offers promising avenues for therapeutic innovation, either through molecular targeting of long non‐coding RNAs or via pharmacological modulation of membrane ion transport. Future investigations should aim to refine NKA‐targeted strategies, assess their safety and efficacy across diverse oncologic settings, and explore their integration within the broader tumor microenvironment to enhance therapeutic precision and overcome resistance mechanisms.

In conclusion, this study provides novel insights into the molecular interactions between LncDARS‐AS1 and ATP1A1 in osteosarcoma progression. These findings advance the mechanistic understanding of osteosarcoma metastasis and establish a foundation for the development of precision therapeutic strategies targeting LncDARS‐AS1 or NKA‐mediated signaling pathways.

## Conflict of Interest

The authors declare no conflict of interest.

## Supporting information



Supporting Information

Supporting Information

## Data Availability

The datasets generated and analyzed during this study are available in the Gene Expression Omnibus (GEO) database under accession numbers GSE14359, GSE14827, GSE21257, GSE32981, and GSE42352. Clinical and gene expression data of osteosarcoma patients were obtained from the Therapeutically Applicable Research to Generate Effective Treatments database (TARGET‐OS). Additional data supporting the findings of this study are available from the corresponding author upon request.
