# Correction to “LncDARS‐AS1 Regulates ATP1A1 Stability and Enhances Na^+^/K^+^ ATPase Activity to Promote Osteosarcoma Metastasis”

**DOI:** 10.1002/advs.74985

**Published:** 2026-03-26

M. Xu, J. Wei, X. Feng, Q. Zhang, J. Chen, X. Wang, X. Zhan, B. Lu, W. Guo, M. Cheng, R. Huang, S. Xu, and C. Zou, “LncDARS‐AS1 Regulates ATP1A1 Stability and Enhances Na^+^/K^+^ ATPase Activity to Promote Osteosarcoma Metastasis,” *Advanced Science* 12, no. 34 (2025): e03486, https://doi.org/10.1002/advs.202503486.

**1. Figures 2G (Bioluminescence imaging)**:

Due to a figure assembly error, representative images in Figure 2G were mislinked and have now been corrected, and the ROI measurements were re‐standardized accordingly. The study's conclusions remain unchanged. The corrected Figure 2G is shown as follows.

Corrected Figure 2G


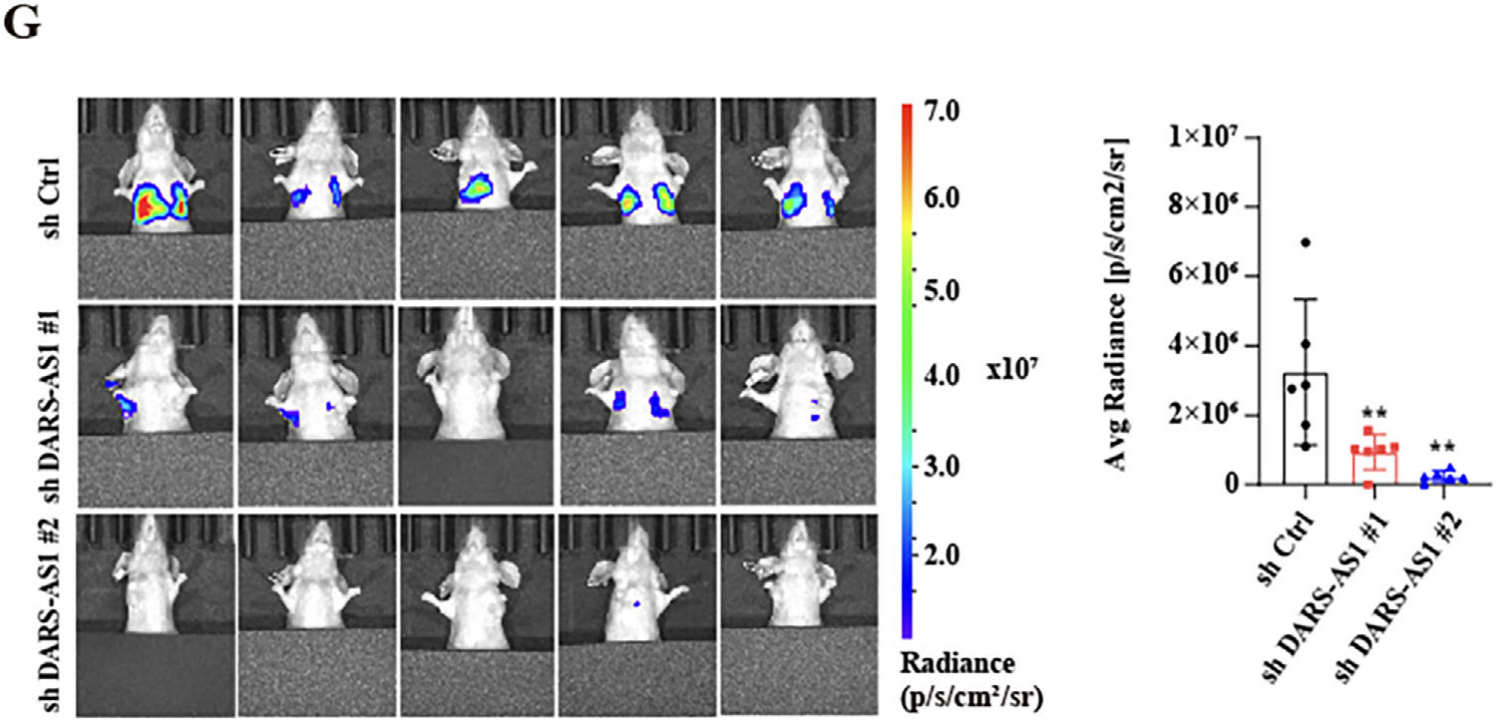


We apologize for this error.

**2. Figures 5F and 5G (Bioluminescence imaging)**:

Several representative orthotopic and lung bioluminescence images were incorrectly assigned during figure assembly. These panels have now been replaced with provenance‐verified original images from the correct experimental groups. The study's conclusions remain unchanged. The corrected Figure 5F,G are shown as follows.

Corrected Figure 5F,G


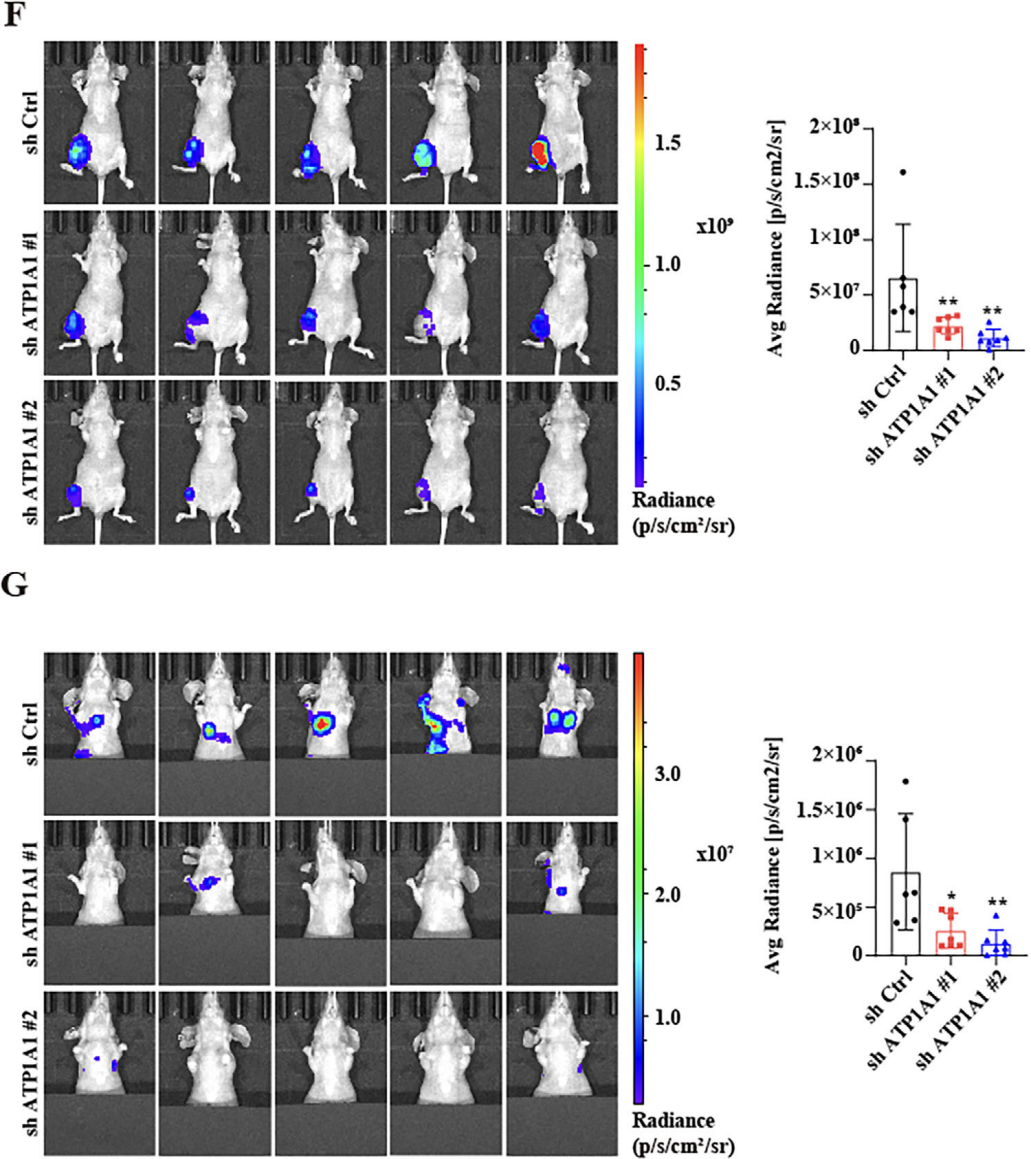


We apologize for this error.

**3. Figure 8G (immunoprecipitation assay)**:

A labeling error occurred in the IP‐ATP1A1 group, where a “+” symbol was incorrectly shown instead of “−.” This labeling has been corrected. The error was limited to labeling only and does not affect the experimental conclusions. The corrected Figure 8G is shown as follows.

Corrected Figure 8G


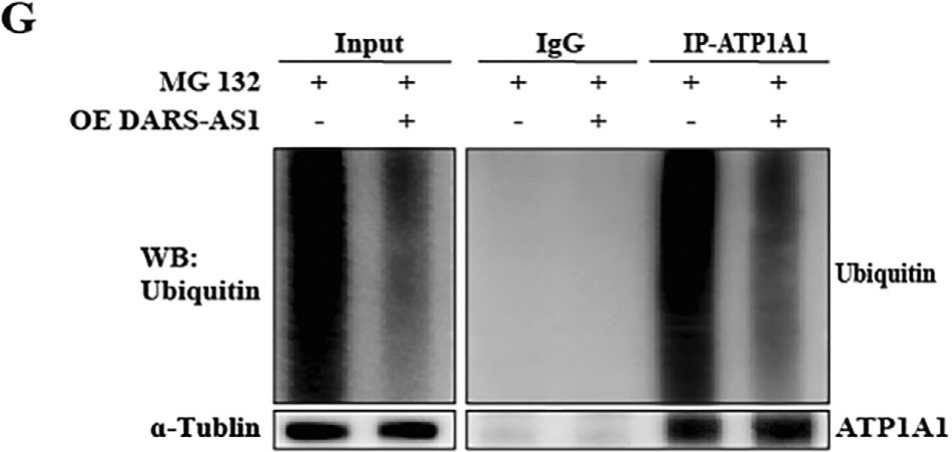


We apologize for this error.

**4. Transwell assay representative images (Supplementary Figures 1E, 1F, and 1L)**:

Representative images in Supplementary Figures 1E, 1F, and 1L were inadvertently misassigned between experimental batches during figure assembly and have now been corrected. The overall conclusions remain unchanged. The corrected Supplementary Figures 1E, 1F, and 1L are shown as follows.

Corrected Supplementary Figures 1E, 1F, and 1L


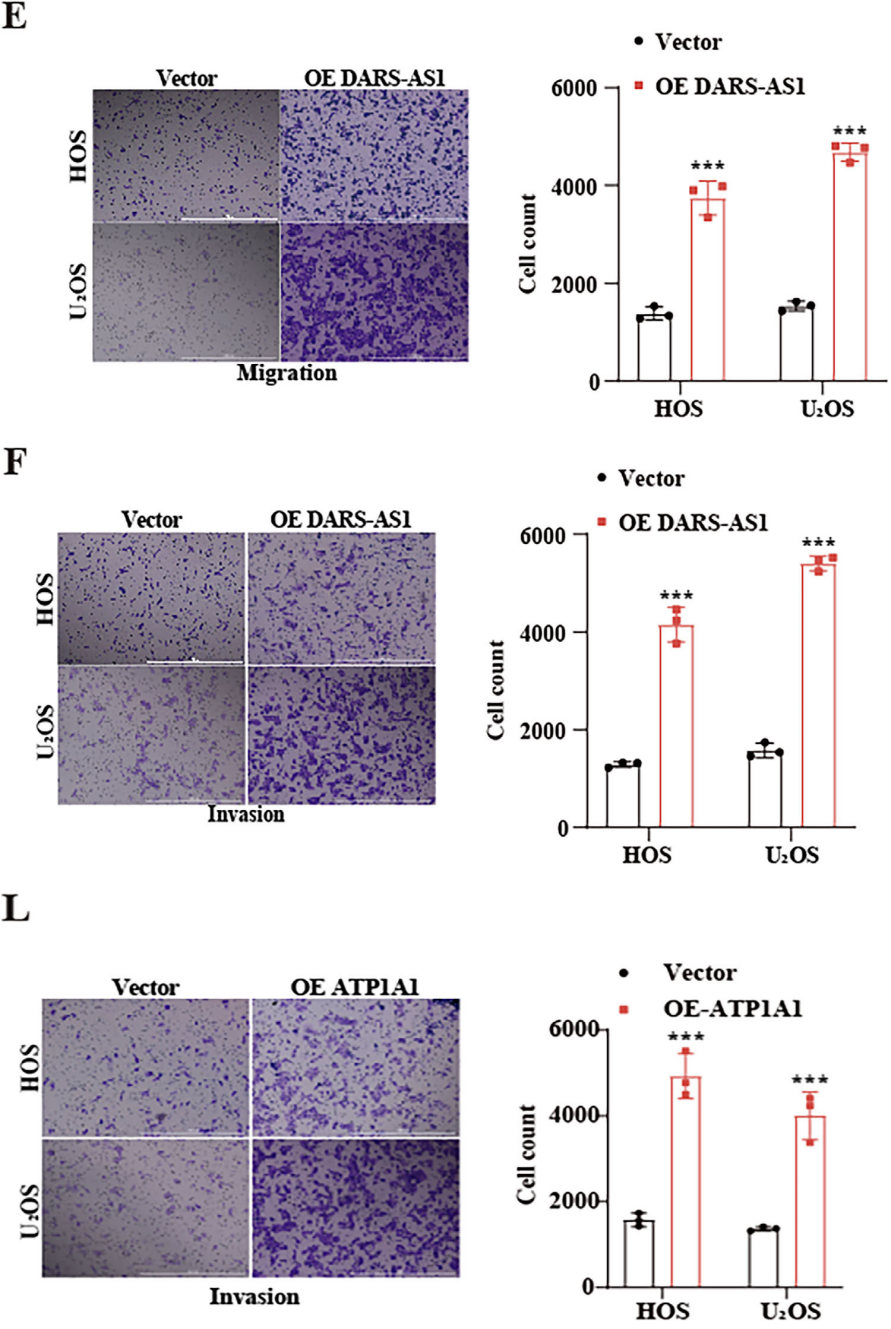


We apologize for this error.

