# Supplementary material for: LncDARS‐AS1 Regulates ATP1A1 Stability and Enhances Na+/K+ ATPase Activity to Promote Osteosarcoma Metastasis
Source: Adv Sci (Weinh). 2025 Jul 15;12(34):e03486. doi: 10.1002/advs.202503486 (PMC12442677; doi:10.1002/advs.202503486)
Supplement: Supplementary file 1 — Supporting Information [file ADVS-12-e03486-s002.docx]

**
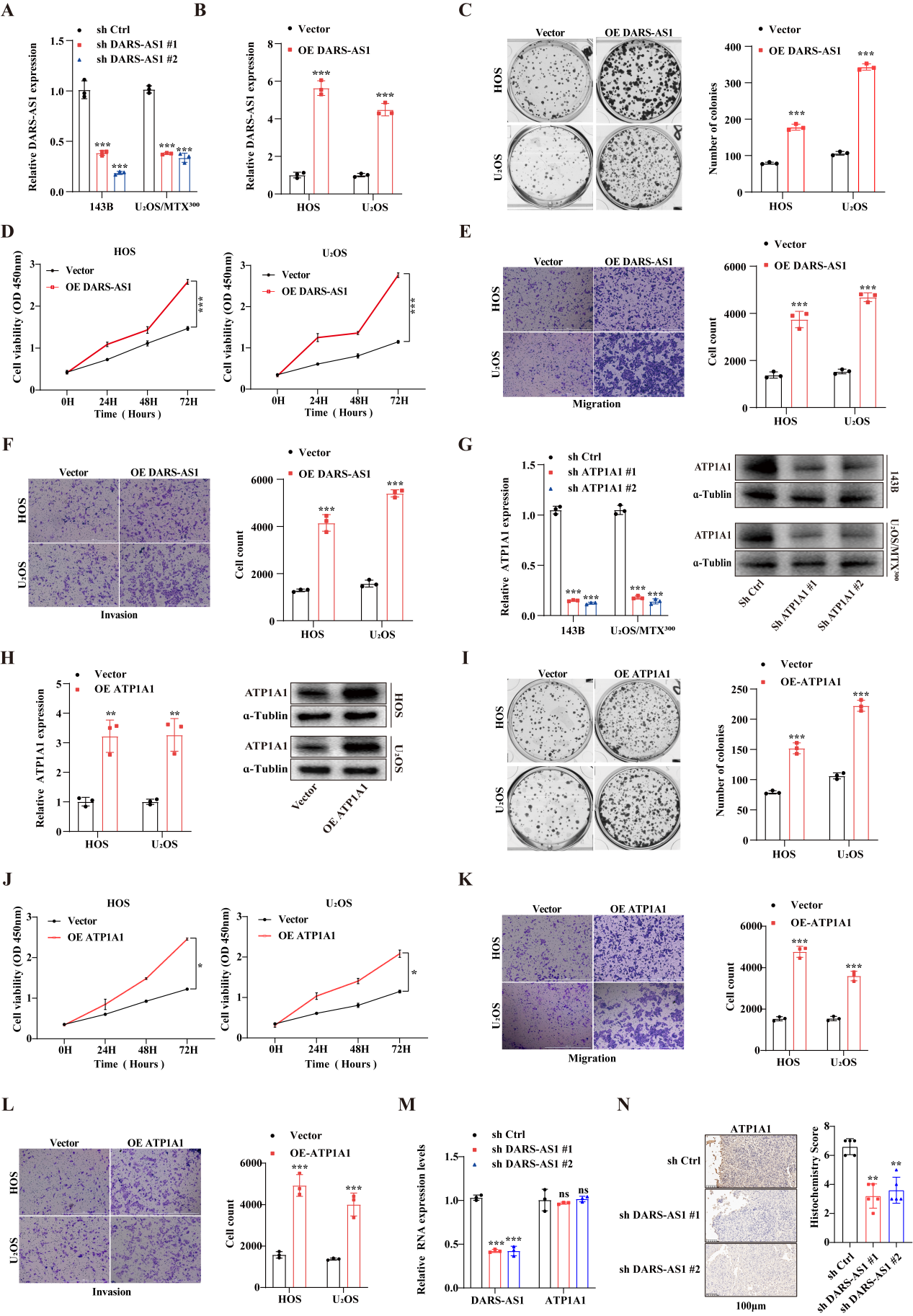
**

**Supplementary Figure S1: Establishment of stable cell lines and functional assays of LncDARS-AS1 and ATP1A1.**

(A)Stable knockdown of LncDARS-AS1 in 143B and U_2_OS/MTX^300^ osteosarcoma cells lines.

(B) Stable overexpression of LncDARS-AS1 in HOS and U_2_OS osteosarcoma cells lines.

(C) Colony formation assays demonstrate increased clonogenic capacity following LncDARS-AS1 overexpression in HOS and U_2_OS cells.

(D) CCK-8 assays show enhanced proliferative activity in LncDARS-AS1 overexpressing HOS and U_2_OS cells.

1. Migration assays indicated significantly increased migration in LncDARS-AS1 overexpressing HOS and U_2_OS cells.
2. Invasion assays showed enhanced invasion ability in LncDARS-AS1 overexpressing HOS and U_2_OS cells.
3. Stable knockdown of ATP1A1 in 143B and U_2_OS/MTX^300^ osteosarcoma cell lines.
4. Stable overexpression of ATP1A1 in HOS and U_2_OS osteosarcoma cell lines.
5. Colony formation assays showed increased colony formation in ATP1A1 overexpressing HOS and U_2_OS cells.

(J) CCK8 assays measured increased proliferation in ATP1A1 overexpressing HOS and U_2_OS cells.

(K) Migration assays showed significantly enhanced migration in ATP1A1 overexpressing HOS and U_2_OS cells.

(L) Invasion assays demonstrate elevated invasive potential in ATP1A1 overexpressing HOS and U_2_OS cells.

(M) Quantitative analysis of RNA extracted from orthotopic tumor tissues in nude mice confirms effective LncDARS-AS1 knockdown.

(N) Immunohistochemical staining of orthotopic tumor sections shows reduced ATP1A1 protein expression following LncDARS-AS1 knockdown in vivo.
