# Supplementary material for: LncDARS‐AS1 Regulates ATP1A1 Stability and Enhances Na+/K+ ATPase Activity to Promote Osteosarcoma Metastasis
Source: Adv Sci (Weinh). 2025 Jul 15;12(34):e03486. doi: 10.1002/advs.202503486 (PMC12442677; doi:10.1002/advs.202503486)
Supplement: Supplementary file 2 — Supporting Information [file ADVS-12-e03486-s001.docx]

**Supplementary Table 1. Reagent or resource source identifier.**

| **REAGENT or RESOURCE** | **SOURCE** | **IDENTIFIER** |
| --- | --- | --- |
| **Experimental models: Cell lines** |  |  |
| 143B | ATCC | CRL-8303 |
| MNNG/HOS | ATCC | CRL-1547 |
| HOS | ATCC | CRL-1543 |
| U_2_OS/MTX^300^ | In our lab | N/A |
| U_2_OS | ATCC | HTB-96 |
| G292 | ATCC | CRL-1423 |
| SJSA-1 | ATCC | CRL-2098 |
| MG63 | ATCC | CRL-1427 |
| SaOS-2 | ATCC | HTB-85 |
| hFOB1.19 | ATCC | CRL-3602 |
| **Sequence information** |  |  |
| Control shRNA | IGE Biotechnology  Guangzhou |  |
| 5'-CACCGTCTTTCAAGGATATGT-3' |  | N/A |
| Sh DARS-AS1#1 | IGE Biotechnology  Guangzhou | N/A |
| 5′- GGCTTATAAGGGACTATATCT-3′ |  |  |
| Sh DARS-AS1#2 | IGE Biotechnology  Guangzhou | N/A |
| 5′- GCATAGAGTCAACGTTGAAGG-3′ |  |  |
| Sh ATP1A1 #1 | IGE Biotechnology  Guangzhou | N/A |
| 5′-CCTGCTGACCTCAGAATCATA-3′ |  |  |
| Sh ATP1A1 #2 | IGE Biotechnology  Guangzhou | N/A |
| 5′-CGGCAGTGATCTAAAGGACAT-3′ |  |  |
| Sh UBQLN4 #1 | IGE Biotechnology  Guangzhou | N/A |
| 5′-GTGGGGTCCGGGATATCCCAA-3′ |  |  |
| Sh UBQLN4 #2 | IGE Biotechnology  Guangzhou | N/A |
| 5′- GCTGCTCAGATGATGGTGAAT-3′ |  |  |
| Sh UBQLN4 #3 | IGE Biotechnology  Guangzhou | N/A |
| 5′- CGGGAACAGTTTGGCAACAAT-3′ |  |  |
| Full-length DARS-AS1 | IGE Biotechnology  Guangzhou | N/A |
| NR_110199.1 |  |  |
| ATP1A1 | IGE Biotechnology  Guangzhou | N/A |
| NM_000701.8 |  |  |
| **PCR primer (Human )** |  |  |
| **DARS-AS1** | RiboBio  Guangzhou | N/A |
| Forward primer (5ʹ–3ʹ) |  |  |
| CCCTAACAGAGTGGTGAGGC |  |  |
| Reverse primer (5ʹ–3ʹ) |  |  |
| CGCTTTCAGAAAACCACCCG |  |  |
| **DARS** | RiboBio  Guangzhou | N/A |
| Forward primer (5ʹ–3ʹ) |  |  |
| GTGGGAGACCATGCAAGCAA |  |  |
| Reverse primer (5ʹ–3ʹ) |  |  |
| AACCGTAGCTCTTCCGTCCT |  |  |
| **ATP1A1** | RiboBio  Guangzhou | N/A |
| Forward primer (5ʹ–3ʹ) |  |  |
| ACAGACTTGAGCCGGGGATTA |  |  |
| Reverse primer (5ʹ–3ʹ) |  |  |
| TCCATTCAGGAGTAGTGGGAG |  |  |
| **UBQLN4** | RiboBio  Guangzhou | N/A |
| Forward primer (5ʹ–3ʹ) |  |  |
| ATTCGGGTCACCGTCAAGAC |  |  |
| Reverse primer (5ʹ–3ʹ) |  |  |
| GCCTTAAACCTCCGGGAGATTT |  |  |
| **GAPDH** | RiboBio  Guangzhou | N/A |
| Forward primer (5ʹ–3ʹ) |  |  |
| GGAGCGAGATCCCTCCAAAAT |  |  |
| Reverse primer (5ʹ–3ʹ) |  |  |
| GGCTGTTGTCATACTTCTCATGG |  |  |
| **U6** | RiboBio  Guangzhou | N/A |
| Forward primer (5ʹ–3ʹ) |  |  |
| ssD0904071006 |  |  |
| Reverse primer (5ʹ–3ʹ) |  |  |
| ssD0904071007 |  |  |
| **DARS-AS1 Probe (ChIRP)** | RiboBio  Guangzhou | N/A |
| NC |  |  |
| TTACGTCAGAAGCGTAGTGC-/3bio |  |  |
| Probe1 |  |  |
| AGCATCCACTTTAAGTCAGG-/3bio(nucleotides 362-381) |  |  |
| Probe2 |  |  |
| GAGTTTGGCCTACTAGAAGC-/3bio (nucleotides 2-21) |  |  |
| Probe3 |  |  |
| GGAAGTGGGTTTCCTCCTTT-/3bio(nucleotides 212-231) |  |  |
| **Truncated versions of DARS-AS1** |  |  |
| **1** |  |  |
| 1-F: cgcttctagtaggccaaactcc |  |  |
| 1-R: ctgtgtccatgtgttctcattgttca |  |  |
| **2** |  |  |
| 2-F: gccgcgtccatgatctcc |  |  |
| 1-R: ctgtgtccatgtgttctcattgttca |  |  |
| **3** |  |  |
| 3-F: tgctttggaaagcactggaaaaatgg |  |  |
| 1-R: ctgtgtccatgtgttctcattgttca |  |  |
| **4** |  |  |
| 1-F: cgcttctagtaggccaaactcc |  |  |
| 4-R: cgatgcccagcgcca |  |  |
| **5** |  |  |
| 1-F: cgcttctagtaggccaaactcc |  |  |
| 5-R: aggctgcgggcgt |  |  |
| **6** |  |  |
| F:AAGAAGACCTAATCGTGGGACT |  |  |
| R:GCTGTGAACCAACATCAGCTC |  |  |
| **Truncated versions of ATP1A1** |  |  |
| **WT-HA** |  |  |
| ATP1A1-F:gactctactagaggatcgctagcgctatggcctttaaggttggacgt |  |  |
| ATP1A1-R:GGCATAATCTGGCACATCATAAGGGTAggatccatagtaggtttccttctccacccag |  |  |
| **∆1-163** |  |  |
| F:gactctactagaggatcgctagcgctatccaagctgctacagaagaggaac |  |  |
| R: GGCATAATCTGGCACATCATAAGGGTAggatccatagtaggtttccttctccacccag |  |  |
| **∆Pfam-E1-E2-ATPase** |  |  |
| F: gctaaaagttcaaagatcatggaatccttcaaaaactgcttagtgaagaacttagaagc |  |  |
| R: gcttctaagttcttcactaagcagtttttgaaggattccatgatctttgaacttttagc |  |  |
| ATP1A1-F:gactctactagaggatcgctagcgctatggcctttaaggttggacgt |  |  |
| ATP1A1-R: GGCATAATCTGGCACATCATAAGGGTAggatccatagtaggtttccttctccacccag |  |  |
| **∆Pfam-Cation-ATPase** |  |  |
| F: gcttgctctgtccagaattgcacccctggatgaggagctgaaaga |  |  |
| R: tctttcagctcctcatccaggggtgcaattctggacagagcaagc |  |  |
| ATP1A1-F:gactctactagaggatcgctagcgctatggcctttaaggttggacgt |  |  |
| ATP1A1-R: GGCATAATCTGGCACATCATAAGGGTAggatccatagtaggtttccttctccaccca |  |  |
| **∆521-799** |  |  |
| F: cctccacggcaaggagcagccactaccactggggactgt |  |  |
| R: acagtccccagtggtagtggctgctccttgccgtggagg |  |  |
| ATP1A1-F:gactctactagaggatcgctagcgctatggcctttaaggttggacgt |  |  |
| ATP1A1-R: GGCATAATCTGGCACATCATAAGGGTAggatccatagtaggtttccttctccacccag |  |  |
| **∆799-1023** |  |  |
| R1: ATCTGGCACATCATAAGGGTAggatccaatgtttgcaataataaatatcaggaacgggg |  |  |
| R2: ATCTGGCACATCATAAGGGTAggatccatagtaggtttccttctccacccagccgccag |  |  |
| R3: cctacccggtagaattatctagattaGGCATAATCTGGCACATCATAAGGGTAggatcc |  |  |
| ATP1A1-F:gactctactagaggatcgctagcgctatggcctttaaggttggacgt |  |  |
| **Experimental models: Organisms** |  |  |
| BALB/c nude mice | VITAL RIVER  Beijing/Ruiye model animal Biotechnology | N/A |

**Supplementary Table 2. The list of top 50 *ChIRP* candidate proteins detected by MS**

| Protein ID | Accession | -10lgP | Avg. Mass | Description |
| --- | --- | --- | --- | --- |
| 81 | sp\|P05023\|AT1A1_HUMAN | 79.63 | 112896 | Sodium/potassium-transporting ATPase subunit alpha-1 OS=Homo sapiens OX=9606 |
| 164 | sp\|P16070\|CD44_HUMAN | 76.27 | 81538 | CD44 antigen OS=Homo sapiens OX=9606 |
| 108 | sp\|P16989\|YBOX3_HUMAN | 75.02 | 40090 | Y-box-binding protein 3 OS=Homo sapiens OX=9606 |
| 94 | sp\|P15311\|EZRI_HUMAN | 70.58 | 69413 | Ezrin OS=Homo sapiens OX=9606 |
| 101 | sp\|P27348\|1433T_HUMAN | 69.85 | 27764 | 14-3-3 protein theta OS=Homo sapiens OX=9606 |
| 100 | sp\|P31946\|1433B_HUMAN | 66.73 | 28082 | 14-3-3 protein beta/alpha OS=Homo sapiens OX=9606 |
| 150 | sp\|P00558\|PGK1_HUMAN | 66.69 | 44615 | Phosphoglycerate kinase 1 OS=Homo sapiens OX=9606 |
| 152 | sp\|P62249\|RS16_HUMAN | 62 | 16445 | 40S ribosomal protein S16 OS=Homo sapiens OX=9606 |
| 177 | sp\|P62750\|RL23A_HUMAN | 61.76 | 17695 | 60S ribosomal protein L23a OS=Homo sapiens OX=9606 |
| 154 | sp\|P0DMV8\|HS71A_HUMAN | 61.54 | 70052 | Heat shock 70 kDa protein 1A OS=Homo sapiens OX=9606 |
| 155 | sp\|P0DMV9\|HS71B_HUMAN | 61.54 | 70052 | Heat shock 70 kDa protein 1B OS=Homo sapiens OX=9606 |
| 135 | sp\|P63104\|1433Z_HUMAN | 60.51 | 27745 | 14-3-3 protein zeta/delta OS=Homo sapiens OX=9606 |
| 157 | sp\|P22626\|ROA2_HUMAN | 57.56 | 37430 | Heterogeneous nuclear ribonucleoproteins A2/B1 OS=Homo sapiens OX=9606 |
| 178 | sp\|P61981\|1433G_HUMAN | 55.83 | 28303 | 14-3-3 protein gamma OS=Homo sapiens OX=9606 |
| 199 | sp\|P62937\|PPIA_HUMAN | 55.35 | 18012 | Peptidyl-prolyl cis-trans isomerase A OS=Homo sapiens OX=9606 |
| 284 | sp\|P09493\|TPM1_HUMAN | 54.29 | 32709 | Tropomyosin alpha-1 chain OS=Homo sapiens OX=9606 |
| 241 | sp\|P62263\|RS14_HUMAN | 53.16 | 16273 | 40S ribosomal protein S14 OS=Homo sapiens OX=9606 |
| 170 | sp\|Q99879\|H2B1M_HUMAN | 52.25 | 13989 | Histone H2B type 1-M OS=Homo sapiens OX=9606 |
| 171 | sp\|Q93079\|H2B1H_HUMAN | 52.25 | 13892 | Histone H2B type 1-H OS=Homo sapiens OX=9606 |
| 172 | sp\|Q99877\|H2B1N_HUMAN | 52.25 | 13922 | Histone H2B type 1-N OS=Homo sapiens OX=9606 |
| 179 | sp\|O60814\|H2B1K_HUMAN | 52.25 | 13890 | Histone H2B type 1-K OS=Homo sapiens OX=9606 |
| 180 | sp\|P62807\|H2B1C_HUMAN | 52.25 | 13906 | Histone H2B type 1-C/E/F/G/I OS=Homo sapiens OX=9606 |
| 181 | sp\|P58876\|H2B1D_HUMAN | 52.25 | 13936 | Histone H2B type 1-D OS=Homo sapiens OX=9606 |
| 182 | sp\|P57053\|H2BFS_HUMAN | 52.25 | 13944 | Histone H2B type F-S OS=Homo sapiens OX=9606 |
| 183 | sp\|Q5QNW6\|H2B2F_HUMAN | 52.25 | 13920 | Histone H2B type 2-F OS=Homo sapiens OX=9606 |
| 184 | sp\|Q99880\|H2B1L_HUMAN | 52.25 | 13952 | Histone H2B type 1-L OS=Homo sapiens OX=9606 |
| 290 | sp\|Q15365\|PCBP1_HUMAN | 47.99 | 37498 | Poly(rC)-binding protein 1 OS=Homo sapiens OX=9606 |
| 255 | sp\|P26373\|RL13_HUMAN | 47.92 | 24261 | 60S ribosomal protein L13 OS=Homo sapiens OX=9606 |
| 257 | sp\|P11021\|BIP_HUMAN | 46.17 | 72333 | Endoplasmic reticulum chaperone BiP OS=Homo sapiens OX=9606 |
| 250 | sp\|Q86YZ3\|HORN_HUMAN | 46.01 | 282389 | Hornerin OS=Homo sapiens OX=9606 |
| 243 | sp\|Q71DI3\|H32_HUMAN | 45.17 | 15388 | Histone H3.2 OS=Homo sapiens OX=9606 |
| 244 | sp\|P84243\|H33_HUMAN | 45.17 | 15328 | Histone H3.3 OS=Homo sapiens OX=9606 |
| 245 | sp\|Q16695\|H31T_HUMAN | 45.17 | 15508 | Histone H3.1t OS=Homo sapiens OX=9606 |
| 246 | sp\|P68431\|H31_HUMAN | 45.17 | 15404 | Histone H3.1 OS=Homo sapiens OX=9606 |
| 261 | sp\|P01116\|RASK_HUMAN | 45.1 | 21656 | GTPase KRas OS=Homo sapiens OX=9606 |
| 306 | sp\|P55884\|EIF3B_HUMAN | 44.88 | 92482 | Eukaryotic translation initiation factor 3 subunit B OS=Homo sapiens OX=9606 |
| 232 | sp\|Q13310\|PABP4_HUMAN | 43.46 | 70783 | Polyadenylate-binding protein 4 OS=Homo sapiens OX=9606 |
| 337 | sp\|P52272\|HNRPM_HUMAN | 41.53 | 77516 | Heterogeneous nuclear ribonucleoprotein M OS=Homo sapiens OX=9606 |
| 316 | sp\|P30101\|PDIA3_HUMAN | 41.39 | 56782 | Protein disulfide-isomerase A3 OS=Homo sapiens OX=9606 |
| 253 | sp\|P21333\|FLNA_HUMAN | 41.16 | 280737 | Filamin-A OS=Homo sapiens OX=9606 |
| 342 | sp\|Q03252\|LMNB2_HUMAN | 40.53 | 69948 | Lamin-B2 OS=Homo sapiens OX=9606 |
| 338 | sp\|Q9NP90\|RAB9B_HUMAN | 39.77 | 22719 | Ras-related protein Rab-9B OS=Homo sapiens OX=9606 |
| 339 | sp\|Q15771\|RAB30_HUMAN | 39.77 | 23058 | Ras-related protein Rab-30 OS=Homo sapiens OX=9606 |
| 277 | sp\|Q14964\|RB39A_HUMAN | 39.77 | 25007 | Ras-related protein Rab-39A OS=Homo sapiens OX=9606 |
| 318 | sp\|Q16658\|FSCN1_HUMAN | 38.44 | 54530 | Fascin OS=Homo sapiens OX=9606 |
| 361 | sp\|P61978\|HNRPK_HUMAN | 38.33 | 50976 | Heterogeneous nuclear ribonucleoprotein K OS=Homo sapiens OX=9606 |
| 260 | sp\|P50991\|TCPD_HUMAN | 38.28 | 57924 | T-complex protein 1 subunit delta OS=Homo sapiens OX=9606 |
| 395 | sp\|P16949\|STMN1_HUMAN | 38.14 | 17302 | Stathmin OS=Homo sapiens OX=9606 |
| 282 | sp\|P29692\|EF1D_HUMAN | 36.75 | 31122 | Elongation factor 1-delta OS=Homo sapiens OX=9606 |
| 359 | sp\|P09382\|LEG1_HUMAN | 35.95 | 14716 | Galectin-1 OS=Homo sapiens OX=9606 |

**Supplementary Table 3. The list of top 50 Co-*IP* candidate proteins detected by MS**

| Protein ID | Accession | -10lgP | Avg. Mass | Description |
| --- | --- | --- | --- | --- |
| 2291 | Q9NRR5 | 40.69 | 63853 | Ubiquilin-4 OS=Homo sapiens OX=9606 GN=UBQLN4 PE=1 SV=2 |
| 241 | P27694 | 40.32 | 68138 | Replication protein A 70 kDa DNA-binding subunit OS=Homo sapiens OX=9606 GN=RPA1 PE=1 SV=2 |
| 1094 | P53680 | 40.09 | 17018 | AP-2 complex subunit sigma OS=Homo sapiens OX=9606 GN=AP2S1 PE=1 SV=2 |
| 944 | Q16891 | 40.08 | 83678 | MICOS complex subunit MIC60 OS=Homo sapiens OX=9606 GN=IMMT PE=1 SV=1 |
| 2114 | P36957 | 39.89 | 48755 | Dihydrolipoyllysine-residue succinyltransferase component of 2-oxoglutarate dehydrogenase complex mitochondrial OS=Homo sapiens OX=9606 GN=DLST PE=1 SV=4 |
| 1145 | Q06203 | 39.31 | 57399 | Amidophosphoribosyltransferase OS=Homo sapiens OX=9606 GN=PPAT PE=1 SV=1 |
| 2107 | Q9NX20 | 39.09 | 28449 | 39S ribosomal protein L16 mitochondrial OS=Homo sapiens OX=9606 GN=MRPL16 PE=1 SV=1 |
| 2199 | O96005 | 38.57 | 76097 | Putative lipid scramblase CLPTM1 OS=Homo sapiens OX=9606 GN=CLPTM1 PE=1 SV=1 |
| 2668 | P30536 | 38.15 | 18828 | Translocator protein OS=Homo sapiens OX=9606 GN=TSPO PE=1 SV=3 |
| 671 | Q14527 | 37.88 | 113928 | Helicase-like transcription factor OS=Homo sapiens OX=9606 GN=HLTF PE=1 SV=2 |
| 875 | Q99986 | 37.36 | 45476 | Serine/threonine-protein kinase VRK1 OS=Homo sapiens OX=9606 GN=VRK1 PE=1 SV=1 |
| 308 | Q15397 | 37.23 | 73584 | Pumilio homolog 3 OS=Homo sapiens OX=9606 GN=PUM3 PE=1 SV=3 |
| 9995 | Q9NRD8 | 37.14 | 175363 | Dual oxidase 2 OS=Homo sapiens OX=9606 GN=DUOX2 PE=1 SV=2 |
| 1438 | O43688 | 36.68 | 32574 | Phospholipid phosphatase 2 OS=Homo sapiens OX=9606 GN=PLPP2 PE=1 SV=1 |
| 888 | P13489 | 36.5 | 49974 | Ribonuclease inhibitor OS=Homo sapiens OX=9606 GN=RNH1 PE=1 SV=2 |
| 7154 | Q15643 | 35.99 | 227584 | Thyroid receptor-interacting protein 11 OS=Homo sapiens OX=9606 GN=TRIP11 PE=1 SV=3 |
| 9820 | Q14257 | 35.74 | 36876 | Reticulocalbin-2 OS=Homo sapiens OX=9606 GN=RCN2 PE=1 SV=1 |
| 9846 | Q5VV42 | 35.66 | 65112 | Threonylcarbamoyladenosine tRNA methylthiotransferase OS=Homo sapiens OX=9606 GN=CDKAL1 PE=1 SV=1 |
| 1607 | Q92616 | 34.97 | 292708 | eIF-2-alpha kinase activator GCN1 OS=Homo sapiens OX=9606 GN=GCN1 PE=1 SV=7 |
| 9830 | Q1L5Z9 | 34.67 | 83654 | LON peptidase N-terminal domain and RING finger protein 2 OS=Homo sapiens OX=9606 GN=LONRF2 PE=1 SV=3 |
| 1666 | Q15011 | 34.14 | 43720 | Homocysteine-responsive endoplasmic reticulum-resident ubiquitin-like domain member 1 protein OS=Homo sapiens OX=9606 GN=HERPUD1 PE=1 SV=1 |
| 897 | P08758 | 32.66 | 35937 | Annexin A5 OS=Homo sapiens OX=9606 GN=ANXA5 PE=1 SV=2 |
| 9931 | Q3T8J9 | 32.65 | 248618 | GON-4-like protein OS=Homo sapiens OX=9606 GN=GON4L PE=1 SV=1 |
| 578 | P19388 | 32.55 | 24551 | DNA-directed RNA polymerases I II and III subunit RPABC1 OS=Homo sapiens OX=9606 GN=POLR2E PE=1 SV=4 |
| 9821 | Q8WU90 | 32.54 | 48603 | Zinc finger CCCH domain-containing protein 15 OS=Homo sapiens OX=9606 GN=ZC3H15 PE=1 SV=1 |
| 3510 | O95202 | 32.39 | 83354 | Mitochondrial proton/calcium exchanger protein OS=Homo sapiens OX=9606 GN=LETM1 PE=1 SV=1 |
| 2202 | Q8NI27 | 31.73 | 182774 | THO complex subunit 2 OS=Homo sapiens OX=9606 GN=THOC2 PE=1 SV=2 |
| 2492 | Q96ME1 | 31.68 | 78919 | F-box/LRR-repeat protein 18 OS=Homo sapiens OX=9606 GN=FBXL18 PE=1 SV=3 |
| 2464 | Q9UKL3 | 30.99 | 222656 | CASP8-associated protein 2 OS=Homo sapiens OX=9606 GN=CASP8AP2 PE=1 SV=1 |
| 9857 | O75396 | 30.95 | 24741 | Vesicle-trafficking protein SEC22b OS=Homo sapiens OX=9606 GN=SEC22B PE=1 SV=5 |
| 1675 | Q9ULW0 | 30.66 | 85653 | Targeting protein for Xklp2 OS=Homo sapiens OX=9606 GN=TPX2 PE=1 SV=2 |
| 323 | Q9NV06 | 30.46 | 51402 | DDB1- and CUL4-associated factor 13 OS=Homo sapiens OX=9606 GN=DCAF13 PE=1 SV=2 |
| 1464 | P05556 | 29.98 | 88415 | Integrin beta-1 OS=Homo sapiens OX=9606 GN=ITGB1 PE=1 SV=2 |
| 9863 | Q53FV1 | 29.34 | 17363 | ORM1-like protein 2 OS=Homo sapiens OX=9606 GN=ORMDL2 PE=1 SV=2 |
| 1157 | O94901 | 28.35 | 87110 | SUN domain-containing protein 1 OS=Homo sapiens OX=9606 GN=SUN1 PE=1 SV=4 |
| 1296 | P48729 | 28.07 | 38915 | Casein kinase I isoform alpha OS=Homo sapiens OX=9606 GN=CSNK1A1 PE=1 SV=2 |
| 988 | P35241 | 27.93 | 68564 | Radixin OS=Homo sapiens OX=9606 GN=RDX PE=1 SV=1 |
| 2285 | P83916 | 27.6 | 21418 | Chromobox protein homolog 1 OS=Homo sapiens OX=9606 GN=CBX1 PE=1 SV=1 |
| 4418 | Q13315 | 27.5 | 350687 | Serine-protein kinase ATM OS=Homo sapiens OX=9606 GN=ATM PE=1 SV=4 |
| 9876 | P15153 | 27.25 | 21429 | Ras-related C3 botulinum toxin substrate 2 OS=Homo sapiens OX=9606 GN=RAC2 PE=1 SV=1 |
| 24 | Q14690 | 26.95 | 208699 | Protein RRP5 homolog OS=Homo sapiens OX=9606 GN=PDCD11 PE=1 SV=3 |
| 1966 | P55061 | 26.33 | 26538 | Bax inhibitor 1 OS=Homo sapiens OX=9606 GN=TMBIM6 PE=1 SV=2 |
| 9879 | P17026 | 26.31 | 25915 | Zinc finger protein 22 OS=Homo sapiens OX=9606 GN=ZNF22 PE=1 SV=3 |
| 2266 | Q12849 | 26.16 | 53126 | G-rich sequence factor 1 OS=Homo sapiens OX=9606 GN=GRSF1 PE=1 SV=3 |
| 595 | P00374 | 26.07 | 21453 | Dihydrofolate reductase OS=Homo sapiens OX=9606 GN=DHFR PE=1 SV=2 |
| 9892 | Q9BZJ0 | 25.8 | 100452 | Crooked neck-like protein 1 OS=Homo sapiens OX=9606 GN=CRNKL1 PE=1 SV=4 |
| 457 | Q6PL18 | 25.72 | 158554 | ATPase family AAA domain-containing protein 2 OS=Homo sapiens OX=9606 GN=ATAD2 PE=1 SV=1 |
| 9860 | Q04721 | 25.71 | 265403 | Neurogenic locus notch homolog protein 2 OS=Homo sapiens OX=9606 GN=NOTCH2 PE=1 SV=3 |
| 261 | Q14684 | 25.63 | 84428 | Ribosomal RNA processing protein 1 homolog B OS=Homo sapiens OX=9606 GN=RRP1B PE=1 SV=3 |
| 637 | Q15008 | 25.41 | 45531 | 26S proteasome non-ATPase regulatory subunit 6 OS=Homo sapiens OX=9606 GN=PSMD6 PE=1 SV=1 |
